# Monosaccharides Dehydration Assisted by Formation of Borate Esters of α-Hydroxyacids in Choline Chloride-Based Low Melting Mixtures

**DOI:** 10.3389/fchem.2020.00569

**Published:** 2020-07-07

**Authors:** Thibaut Istasse, Vincent Lemaur, Gwénaëlle Debroux, Lauris Bockstal, Roberto Lazzaroni, Aurore Richel

**Affiliations:** ^1^Laboratory of Biomass and Green Technologies, University of Liege—Gembloux Agro-Bio Tech, Gembloux, Belgium; ^2^Laboratory for Chemistry of Novel Materials, Materials Research Institute, University of Mons, Mons, Belgium

**Keywords:** low-transition-temperature mixtures, 5-hydroxymethylfurfural, glucose, choline chloride, boric acid

## Abstract

The synthesis of 5-hydroxymethylfurfural (5-HMF) and 2-furfural (2-F) by hexoses and pentoses dehydration is considered as a promising path to produce materials from renewable resources. Low-transition-temperature mixtures (LTTMs) enable selective (>80%) dehydration of ketoses to furanic derivatives at moderate temperature (<100°C). However, aldoses dehydration generally requires higher temperatures and an isomerization catalyst. Chromium trichloride has been reported as one of the most efficient catalyst but its kinetic inertness could limit its performances below 100°C. Consequently, we investigate herein boric acid catalysis of aldoses dehydration in LTTMs based on choline halides and organic acids at 90°C. The limited activity of boric acid regarding furanic compounds synthesis (e.g., 5% 5-HMF yield and 23% glucose conversion after 1 h at 90°C with maleic acid) can be enhanced through tetrahydroxyborate esters (THBE) formation with α-hydroxyacids (e.g., 19% 5-HMF yield and 61% glucose conversion after 1 h at 90°C). THBE formation is however associated with H_3_O^+^ generation favoring the appearance of side products (humins). We demonstrate that boric acid catalysis is not straightforward and that the use of THBE under moderate acidity should be further investigated to limit humins formation and promote furanic derivatives synthesis.

## Introduction

Synthesis of renewable platform chemicals from biomass carbohydrates is considered as a milestone in the development of an efficient use of renewable resources. Among these chemicals, 5-hydroxymethylfurfural (5-HMF) and 2-furfural (2-F), resulting from the dehydration of hexoses (e.g., D-fructose, D-glucose) and pentoses (D-xylulose, D-xylose) respectively, receive particular attention (Rosatella et al., 2011; Van Putten et al., 2013b). Both platform chemicals possess numerous applications in material synthesis including plastics, thermoset resins, pharmaceuticals, and fuels (Rosatella et al., 2011; Eseyin and Steele, 2015). Their production was intensively investigated and high temperature processes (>150°C) were generally required to achieve acceptable selectivity of the reaction (Watanabe et al., 2005; Chheda et al., 2007; Mittal et al., 2017). However, research is currently turned toward eco-friendly and low-temperature processes. In this sense, the use of ionic liquids (ILs) was studied in order to perform dehydration of monosaccharides to 5-HMF and 2-F at moderate temperature (100°C) with high selectivity (>80%) (Moreau et al., 2006; Qi et al., 2008; Simeonov et al., 2012).

ILs have been highlighted as promising solvents due to their tunable properties resulting from the mixing of different cationic and anionic species (Lima et al., 2009; Van Osch et al., 2017). ILs exhibit major advantages such as low vapor pressure, lower temperature required for selective reactions, and potential recyclability. Nevertheless, most of them remain expensive and their synthesis can require several reaction and purification steps (Van Osch et al., 2017). Moreover, constitutive cations like pyridinium and imidazolium can be toxic as they may inhibit crucial enzymes like acetylcholinesterase, which plays an important role in nerve response and function (Thuy Pham et al., 2010). Besides ILs, deep eutectic solvents (DES) are another type of low-transition-temperature mixtures (LTTMs) which has been investigated. Their components are cheap, abundant and less hazardous than ionic liquids and include in particular monosaccharides, organic acids, choline chloride, amino acids, and water (Dai et al., 2013; Paiva et al., 2014; Radošević et al., 2015; Durand et al., 2016; Van Osch et al., 2017). LTTMs are obtained by mixing and heating together two or three of the aforementioned components in proper ratio. This very simple synthesis process enables a 100% carbon efficiency (Zhang et al., 2012).

Istasse et al. (2018) demonstrated the efficiency of LTTM's composed of choline chloride and organic acids to perform D-fructose dehydration to 5-HMF at 60°C. However, it is worth noting that even though the synthesis of furanic derivatives from ketoses (e.g., D-fructose, D-xylulose) is straightforward, their production from aldoses (e.g., D-glucose, D-xylose) remains challenging. Selective and low-cost dehydration process of those abundant hexoses has still to be developed (Rosatella et al., 2011; Van Putten et al., 2013b).

**Figure 1:**
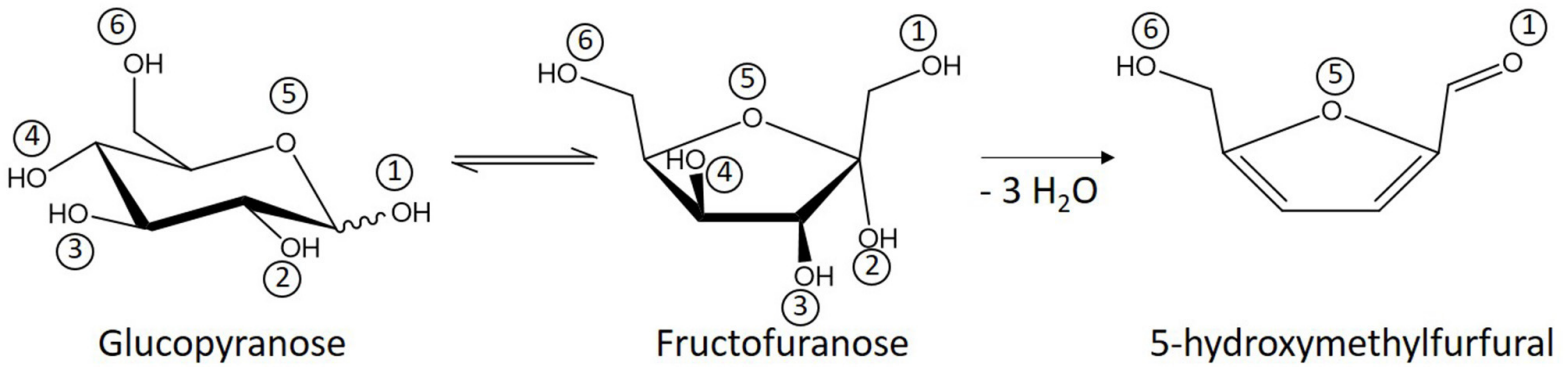
Glucose conversion to 5-hydroxyméthylfurfural (with labeling of oxygen atoms).

Contrarily to ketoses, aldoses cannot be directly dehydrated to furanic derivatives. They first have to undergo isomerization to ketoses or ring contraction, which are mechanisms that are less energetically favored compared to side-reactions including polymerization reactions (Harris and Feather, 1975; Yang et al., 2012; Enslow and Bell, 2015). In order to improve 5-HMF and 2-F formation, isomerization catalysts are added to the reaction medium (Figure 1). CrCl_3_ is generally reported as the most efficient catalyst as it promotes D-glucose isomerization to D-fructose by coordination of the metallic center to the monosaccharide, facilitating the required hydride shift (Binder et al., 2010; Li et al., 2014). However, trivalent chromium is kinetically inert, which means that high temperatures are required to introduce new chemical species in the coordination sphere (Bali et al., 2012). This could limit its potential for reaction catalysis below 100°C. As an alternative, boric acid has been studied as isomerization catalyst. This cheap and abundant non-metallic Lewis acid favors isomerization through an enediol mechanism rather than through hydride shift (Stahlberg et al., 2011). However, its performances in aldoses dehydration catalysis are mitigated (10–40% yield) compared to the results obtained with metallic catalysts (80% yield with CrCl_3_; Stahlberg et al., 2011; Hu et al., 2012).

The aim of this work is to better understand the mechanism of action of boric acid as catalyst in order to explain his limited performances compared to metallic catalyst as well as the possibilities of improvement. Consequently, we explored 5-HMF and 2-F formation from monosaccharides in LTTMs composed of choline chloride, boric acid and organic acids. Firstly, the dehydration of several hexoses and pentoses is performed in a mixture of choline chloride/maleic acid with and without boric acid. Then dehydration of D-glucose is further investigated in mixtures of choline chloride/boric acid combined with different organic acids in an attempt to identify an effect of the organic acid structure (number of carboxylic acid functions, carbon chain length, and presence of additional hydroxyl moieties). The role of halide anions is explored by comparing mixtures with choline chloride, choline bromide and choline iodide. 2D HSQC NMR (^1^H-^13^C) analyses are performed to confirm the production of 5-HMF and identify other soluble side-products. A reaction mechanism is proposed for the conversion of D-glucose to 5-HMF based on experiments and Density Functional Theory (DFT) calculations. The formation of undesirable polymeric substances called humins was also investigated through experiments with different LTTMs, infrared spectroscopy and 2D HSQC NMR (^1^H-^13^C) analyses.

## Materials and Methods

### Chemicals Purities

D-(-)-fructose (99%), choline chloride (99%), maleic acid (99%), *m*-hydroxybenzoic acid (99%), and L-(+)-tartaric acid (>99%) were purchased at Acros Organics. D,L-malic acid (>99%), *o*-hydroxybenzoic acid (>99.0%), *p*-hydroxybenzoic acid (>99%), 3,4-dihydroxybenzoic acid (>97.0%), 1,2-dihydroxybenzene (>99%), D-(+)-xylose (>99%), and 5-HMF (>99%) were acquired from Sigma-Aldrich. Succinic acid (99%) and D,L lactic acid (>88%) were obtained from Fischer Chemical. Boric acid (99.5–100.5%) and 2-F (>98%) were purchased at Merck. Formic acid (99%) was acquired from Biosolve LTD. Acetic acid (99-100%), citric acid (99.8%), and anhydrous D-(+)-Glucose (GPR Rectapur) were obtained from VWR Chemicals. D-(+)-mannose (>99%) and D-(-)-arabinose were purchased at Fluka. Anhydrous oxalic acid (98%) and choline iodide (98%) were obtained from Alfa Aesar. Benzoic acid (>99.5%) was purchased at Roth. Choline bromide (>98.0%) and glycolic acid (>98.0%) were obtained from TCI Chemicals. All chemicals were used as received. Each LTTMs was stored in a sealed vessel directly after preparation.

### Dehydration of Monosaccharides in Choline Chloride/Maleic Acid

A mixture of choline chloride and maleic acid (molar ratio: 2/1) was prepared by mixing both solid components in a closed vessel at 90°C until a liquid phase was obtained. The choice of both components and their ratio is based on previous work (Istasse et al., 2018). Except if specified, all reactions were carried out under air.

Preliminary tests were performed in sealed glass tubes by adding 2 g of this mixture (corresponding to 10 mmol of choline chloride and 5 mmol of maleic acid) to hexoses (0.56 mmol) or pentoses (0.56 mmol). Mixtures were homogenized prior to the test. Samples were then heated at 90°C in a water bath with an agitation of 210 rpm. After the treatment, mixtures were immediately dissolved with water, filtered on 0.45 μm syringe filter and stored at −20°C.

For the preliminary tests with boric acid, a mixture of choline chloride, maleic acid, and boric acid was prepared (molar ratio: 10/5/1). For dehydration tests, 2 g of the LTTMs were again added to 0.56 mmol of hexose or pentose, which correspond to 9.8 mmol of choline chloride, 4.9 mmol of maleic acid and 1 mmol of boric acid in each tube.

### Comparison of Organic Acids in Mixtures With Choline Chloride and Boric Acid for the Dehydration of D-Glucose

Compared to the preliminary experiments, the ratio between boric acid and the organic acid in LTTMs was rapidly optimized regarding 5-HMF yield. Considering the large number of organic acids under consideration, LTTMs were directly prepared in glass tubes by homogenizing 0.56 mmol of D-glucose (0.56 mmol) with choline chloride (10 mmol), the organic acid (2.5 mmol), and boric acid (2.5 mmol). Samples were heated at 90°C during 1 h at 210 rpm. During the first 5 min of the treatment, samples were again thoroughly homogenized to ensure the rapid formation (about 5 min) of the liquid phase. After the treatment, mixtures were immediately dissolved with water, filtered on 0.45 μm syringe filter and stored at −20°C. The addition of water cools down the reaction medium and inhibit monosaccharides dehydration as demonstrated in previous work (Istasse et al., 2018). The same procedure was followed for the tests with benzoic acid derivatives.

### Inhibition of D-Glucose Dehydration by Addition of 1,2-Dihydroxybenzene

Additional tests were performed to identify a potential inhibition of the reaction by other molecules with a vicinal diol moiety. D-glucose (0.56 mmol) was dehydrated in LTTMs prepared as described in Table 1. Unlike the other LTTMs mentioned in this work, 10 wt% water was added and these assays were performed at 100°C to ensure the formation of a homogeneous liquid phase.

**Table 1:** Organic acids and phenol derivatives added to choline chloride (10 mmol) and boric acid (2.5 mmol) for LTTMs preparation.

**Assay**	**Organic acid (2.5 mmol)**	**Phenol and hydroxyphenol (2.5 mmol)**
1	Benzoic acid	1,2-dihydroxybenzene
2	/	Phenol
3	/	1,2-dihydroxybenzene
4	*o*-hydroxybenzoic acid	Phenol
5	*o*-hydroxybenzoic acid	1,2-dihydroxybenzene
6	*o*-hydroxybenzoic acid	/

### Dehydration of Monosaccharides in Choline Chloride/Boric Acid/Glycolic Acid

0.56 mmol of hexoses or pentoses were heated at 90°C in a mixture of choline chloride/boric acid/glycolic acid (molar ratio: 4/1/1) during 1 h at 210 rpm. The LTTMs was prepared beforehand. Samples were prepared as previously mentioned.

### Effect of Choline Halides on 5-HMF Formation

Choline chloride, bromide or iodide (10 mmol) was mixed with boric acid (2.5 mmol), glycolic acid (2.5 mmol) and D-glucose (0.56 mmol), and heated at 90°C during 1 h at 210 rpm following the same protocol than for the other experiments.

### Acidity Comparison Between LTTMs

Using a UV-1800 Shimadzu spectrophotometer, the absorbance of different mixtures of choline chloride (20 mmol), boric acid (5 mmol), and organic acid (5 mmol) containing 13 wt% water and 50 μL of thymol blue in ethanol (0.05 g/10 ml) was measured at 548 nm. The presence of water ensures the formation of a homogeneous liquid mixture during the absorbance measurement at ambient temperature. To perform the tests with choline chloride/H_3_BO_3_ (molar ratio: 2/1) with or without hydrochloric acid, HCl (35.7%) was added to a 3 g mass of mixture to reach the desired concentration. Different amounts of water were added for each HCl concentration in order to keep the total water content at 13 wt%. 50 μL of thymol blue in ethanol (0.05 g/10 ml) were again added before homogenization and absorbance measurement. The tests with different HCl concentrations were performed to confirm the increase of the absorbance at 548 nm with the acidity (see Supplementary Table 1).

### Analyses of 5-HMF and Monosaccharides

Monosaccharides were quantified by high performance liquid chromatography (HPLC) using a Ca Rezex RPM Monosaccharides column heated at 80°C with a water flow of 0.6 mL/min. Detection was performed with an evaporative light scattering detector (40°C, gas flow of 0.8 L/min). 5-HMF and 2-F were separated on a HPX-87H Aminex column heated at 45°C using a 5 mM H_2_SO_4_ aqueous solution at a 0.6 mL/min flow. UV detection was used to measure 5-HMF and 2-F concentrations at 284 nm. Monosaccharide conversion, 5-HMF/2-F yield and selectivity were calculated as follows:

$$\begin{array}{l} Residual\;monosaccharides\;\left(\%\right)=\\ \;\frac{Final\;moles\;of\;monosaccharides}{Initial\;moles\;of\;monosaccharides}\;\times 100\\ Monosaccharides\;conversion\;\left(\%\right)=\\ \;100-Residual\;monosaccharides\\ 5-HMF\;or\;2-F\;yield\;\left(\%\right)=\\ \;\frac{Moles\;of\;5-HMF\;or\;2-F}{Initial\;moles\;of\;monosaccharides}\;\times 100\\ Selectivity\;\left(\%\right)=\\ \;\frac{\left(5-HMF\;or\;2-F\;yield\right)}{Monosaccharide\;conversion}\;\times 100 \end{array}$$

### NMR Analyses

A LTTMs composed of choline chloride, boric acid and oxalic acid (molar ratio 4/1/1) was prepared with 10 wt% of deuterated water. Mixtures of LTTMs and D-glucose (20–40 wt%) were then heated at 80°C during 10 or 60 min to easily distinguish reaction substrate from products. The high load of D-glucose enabled a better discrimination of monosaccharides and choline chloride signals. Samples without D-glucose were also prepared to rapidly identify LTTMs signals.

The reaction was quenched by addition of deuterated water (12.5 ml of water/g of mixture) containing an internal standard [0.75 wt% of 3-(trimethylsilyl)propionic-2,2,3,3 d4 acid sodium salt] and the resulting NMR samples solutions were filtered (0.45 μm).

2D HSQC (^1^H-^13^C) NMR spectra of these solutions were recorded at 298 K on a Bruker Ultrashield 700 Plus equipment operating at 700 MHz for ^1^H and 175 MHz for ^13^C. All NMR experiments were performed using a triple resonance inverse-probe. The datasets were acquired with 2,048 and 512 data points for the f2 (^1^H) and f1 (^13^C) dimensions, with spectral widths of 11,161 and 38,463 Hz, respectively. Four scans were performed and the relaxation delay was 2 s.

### Density Functional Theory Calculations

The ground-state geometries of all molecules were optimized at the Density Functional Theory (DFT) level using the B3LYP functional and a 6-31G^**^ basis set. The PCM (Polarizable Continuum Model; Tomasi et al., 2005) scheme was coupled to all DFT calculations to account for solvent (dichloromethane) effects. Within this model, the solute is embedded in a shape-adapted cavity surrounded by the solvent implicitly described by a dielectric continuum that is characterized by a dielectric constant. The dielectric constant of dichloromethane (ε = 8.93) was used in this work following the approach of Stahlberg et al. (2011) for their study of D-glucose dehydration in ionic liquids containing boric acid. The literature about LTTMs dielectric constant being scarce, this choice enables comparison between both works to obtain mechanistic insights. For each optimized geometry, a normal-mode analysis was performed for the thermochemical analysis (using a temperature of 298.15 K) and it was verified that an energy minimum was obtained. The reported Gibbs free energies were then estimated as the energy difference between the sum of the energies of the isolated products and the sum of the energies of the isolated reactants. All the DFT calculations were performed with the Gaussian16 package (Frisch et al., 2016).

### Recovery and Analyses of Humins

The generated humins were recovered by centrifugation (2,100 g, 10 min) after treatment and dissolution of the LTTMs and dried at 105°C. Dried humins were analyzed by infrared spectroscopy using a Bruker VERTEX 70 FT-IR device. Dried humins were also solubilized in deuterated DMSO for 2D HSQC (^1^H-^13^C) NMR analyses at 298 K on a Bruker Ultrashield 700 Plus equipment operating at 700 MHz for ^1^H and 175 MHz for ^13^C. All NMR experiments were performed using a triple resonance inverse-probe. The datasets were acquired with 1,024 and 256 data points for the f2 (^1^H) and f1 (^13^C) dimensions, with spectral widths of 11,161 and 38,463 Hz, respectively. Eight scans were performed and the relaxation delay was 1.5 s.

## Results and Discussion

### Dehydration of Monosaccharides in LTTMs

**Figure 2:**
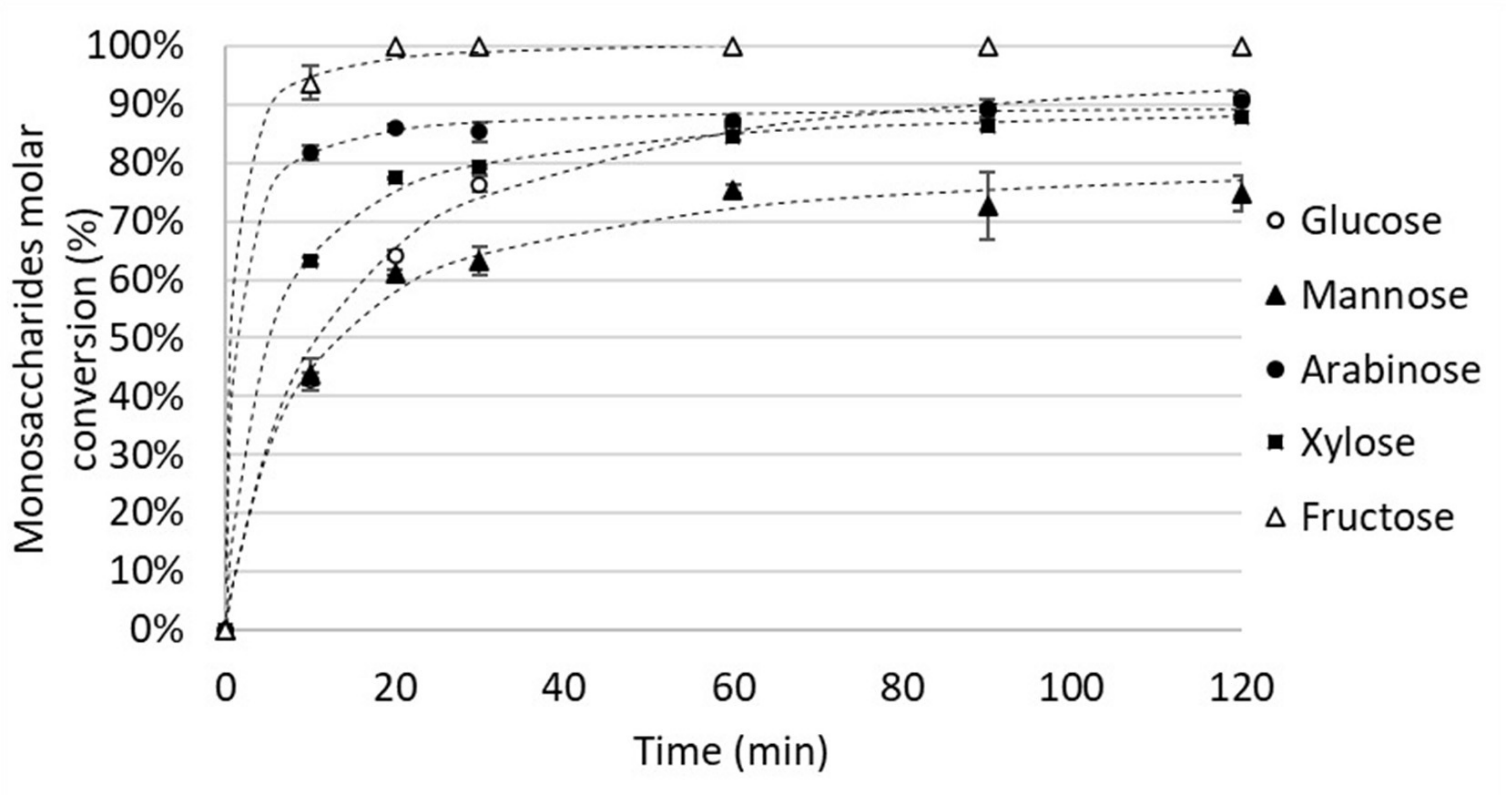
Evolution of hexoses (0.56 mmol) and pentoses (0.67 mmol) molar conversion (%) in a mixture of choline chloride (10 mmol) and maleic acid (5 mmol) at 90°C.

In a preliminary experiment, several hexoses (D-fructose, D-glucose and D-mannose), and pentoses (D-xylose and D-arabinose) were heated during 2 h in a mixture of choline chloride and maleic acid at 90°C. Although maleic acid is known to efficiently dehydrate D-fructose to 5-HMF (Istasse et al., 2018), this attempt did not lead to the appearance of 5-HMF from D-glucose and D-mannose or 2-F from D-arabinose. Only 2.26 ± 0.04% of 2-F was generated from D-xylose. As expected, large amounts of 5-HMF were produced from D-fructose (71.43 ± 1.05%). Conversion of monosaccharides in the LTTM is depicted in Figure 2 (5-HMF and 2-F yields evolution with time are provided in Supplementary Figure 1). The conversion of all monosaccharides is above 70% in less than an hour at 90°C. D-fructose, a ketose, is converted faster than the other monosaccharides (aldoses) as suggested by the work of Van Putten et al. (2013a). The selectivity of the dehydration of aldoses to 5-HMF and 2-F is however insignificant. Esterification of monosaccharides with maleic acid is a possible side-reaction. Esterification between citric acid and glycerol has been reported to occur even at 90°C (Halpern et al., 2014). Consequently, a similar reaction with monosaccharides seems likely to occur in mixtures of organic acids and choline chloride. It could explain the observed plateau trends in aldoses conversion (Figure 2) assuming they are in equilibrium with their esters. Fructose, being easily dehydrated to 5-HMF, rapidly achieves 100% conversion.

After addition of boric acid to the choline chloride/maleic acid mixture, 5-HMF and 2-F were produced from all the tested monosaccharides, as described in Table 2. 5-HMF yields of 55.72 ± 0.98%, 6.54 ± 0.67%, and 4.02 ± 0.15% were achieved from D-fructose, D-glucose and D-mannose, respectively. 2-F yields of 9.56 ± 1.42% and 4.80 ± 0.06% were obtained from D-xylose and D-arabinose. The selectivity of the conversion of those monosaccharides to furan derivatives reached 55.72 ± 0.98 (D-fructose), 11.88 ± 1.87 (D-glucose), 5.35 ± 0.21 (D-mannose), 16.71 ± 2.63 (D-xylose), and 6.92 ± 0.20% (D-arabinose). The reaction selectivity increased for all the aldoses in the presence of boric acid but the production of 5-HMF from D-fructose, a ketose, was more selective in the absence of boric acid (71.43 ± 1.05%). Considering that boric acid enables the reversible isomerization of D-glucose to D-fructose, this observation is not surprising and could be a first clue that D-glucose conversion to 5-HMF proceeds indeed through isomerization to D-fructose in the tested conditions. The presence of boric acid opens a new reaction path for D-fructose which consequently decreases the selectivity for 5-HMF. This point is further discussed after DFT calculations and additional experiments.

**Table 2:** Monosaccharides conversion and 5-HMF and 2-F yields/selectivity obtained after treatment of several hexoses (0.56 mmol) and pentoses (0.67 mmol) in a mixture of choline chloride (10 mmol) and maleic acid (5 mmol) with or without boric acid (1.08 mmol) during 1 h at 90°C.

**Monosaccharides**	**No boric acid**			**Boric acid**		
	**Conversion (%)**	**Yield (%)**	**Selectivity (%)**	**Conversion (%)**	**Yield (%)**	**Selectivity (%)**
Glucose	86.41 ± 0.94	<1	<2	55.37 ± 3.43	6.54 ± 0.67	11.88 ± 1.87
Fructose	100.00 ± 1.00	71.43 ± 1.03	71.43 ± 1.03	100.00 ± 1.00	55.72 ± 0.98	55.72 ± 0.98
Mannose	75.52 ± 0.67	<1	<2	75.12 ± 0.41	4.02 ± 0.15	5.35 ± 0.21
Xylose	84.56 ± 0.65	1.91 ± 0.05	2.26 ± 0.04	57.28 ± 0.77	9.56 ± 1.42	16.71 ± 2.63
Arabinose	87.24 ± 0.32	<1	<2	69.33 ± 2.27	4.80 ± 0.06	6.92 ± 0.20

*5-HMF is the dehydration product obtained from glucose, fructose and mannose. 2-furfural is the dehydration product obtained for xylose and arabinose*.

**Figure 3:**
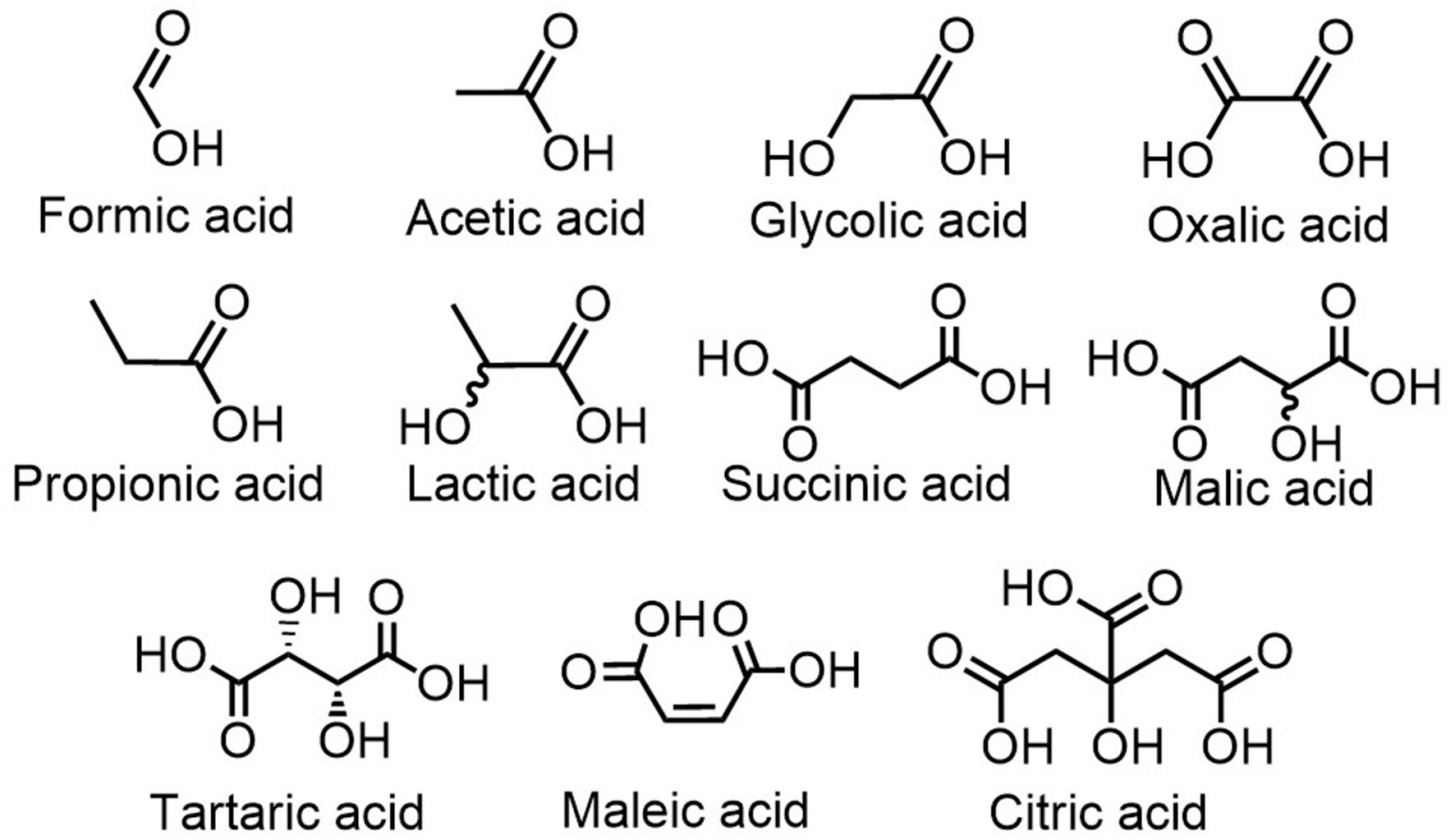
Organic acids used as LTTMs components for the first set of experiments.

For the next set of experiments, the effect of the organic acid structure was investigated. LTTMs were prepared by mixing each acidic component presented in Figure 3 with boric acid and choline chloride (molar ratio: 1/1/4).

**Figure 4:**
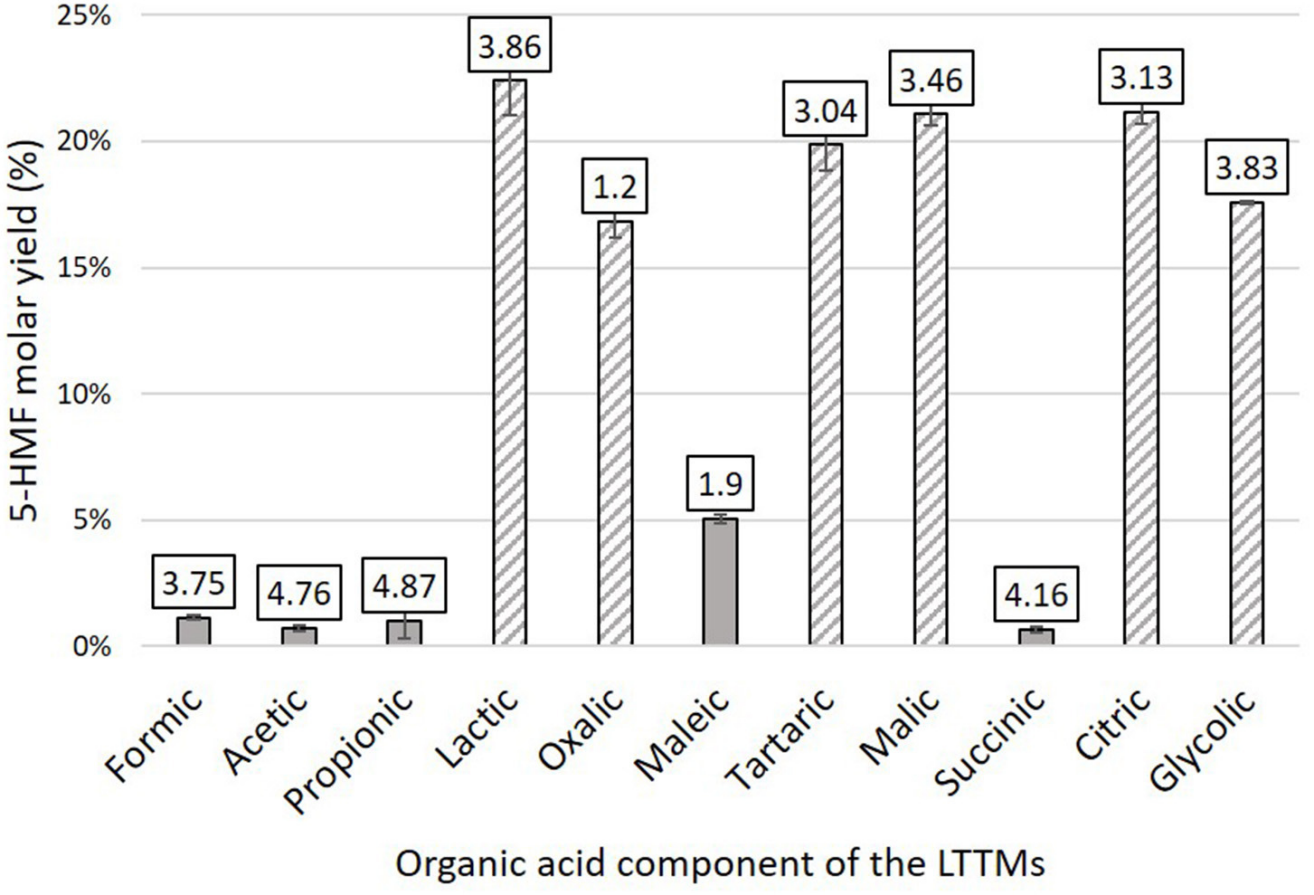
5-HMF yields obtained from D-glucose (0.56 mmol) in mixtures of choline chloride (10 mmol), organic acid (2.5 mmol), and boric acid (2.5 mmol) after 1 h at 90°C, 210 rpm. The pKa of each organic acid component is depicted above each bar. Hatched bars correspond to α-hydroxyacids.

The 5-HMF yields obtained from D-glucose after 1 h at 90°C are described in Figure 4 (The corresponding conversion and selectivity are provided in Supplementary Figures 2, 3). Noticeable differences were observed between acids: some organic acids lead to 5-HMF yields above 15% (hatched bars, Figure 4) while 5-HMF yields lower than 6% are achieved with the others (gray bars, Figure 4). Strikingly, large amounts of humins were generated with all the acids allowing significant 5-HMF formation. This trend was also observed for the conversion of the monosaccharide: more than 60% conversion was achieved in the presence of lactic, oxalic, tartaric, malic, citric, or glycolic acids while <30% of D-glucose was converted in the presence of formic, acetic, propionic, maleic, or succinic acids. Considering selectivity for 5-HMF, the same trend was observed except for the selectivity of maleic acid (22.33 ± 2.10%), which was comparable to those of lactic, oxalic, tartaric, malic, citric, and glycolic acids (18–28%). The best selectivity was obtained with lactic and glycolic acids (28.42 ± 0.27 and 26.10 ± 0.11%, respectively). A control test was performed in the same conditions with a mixture of choline chloride and boric acid (molar ratio: 2/1). Only 16.19 ± 2.32% of D-glucose was converted and a 5-HMF yield of 3.24 ± 0.07% was reached (corresponding to a 20.30 ± 2.70% selectivity).

In a first attempt to understand those results, the pKa of the tested organic acids in aqueous solution were compared (displayed in Figure 4). In LTTMs, the dissociation constant of acids could differ from the aqueous medium. Abbott et al. (2018) studied the Brønsted acidity of several organic acids (oxalic, salicylic, succinic, citric, benzoic, lactic, propanoic, and acetic acids) in LTTMs composed of choline chloride and ethylene glycol, glycerol, or urea. The authors demonstrated that these organic acids were only slightly less dissociated in LTTMs, with pKa values only 0.2–0.5 higher than in water (Abbott et al., 2018). The pKa values in water seem therefore appropriate to compare the acidity of the LTTMs prepared in this work. However, no relationship seems to emerge between the acid pKa and the observed 5-HMF yields. By comparing the chemical structures of the organic acids, it appeared that all α-hydroxyacids allowed a faster and more selective reaction.

### Investigations About Dehydration Catalysis by Boric Acid and α-Hydroxyacids

**Figure 5:**
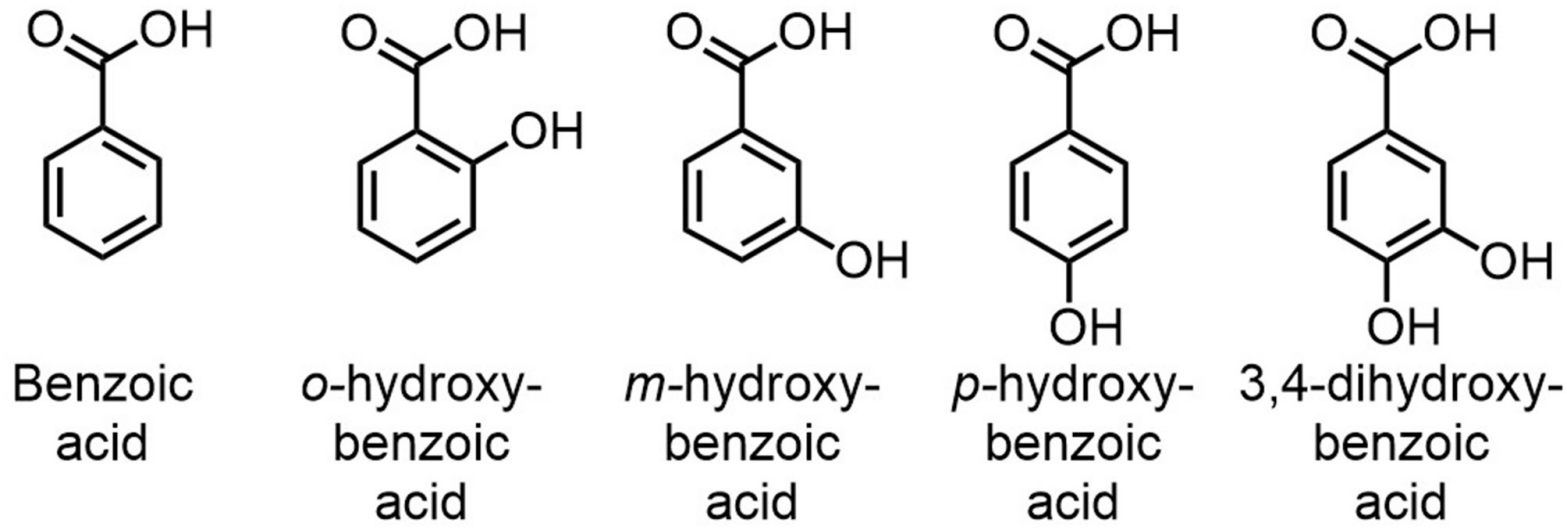
Derivatives of benzoic acid used as LTTMs components for the second set of experiments.

Additional tests were consequently performed to confirm and identify the role of the proximity between the –OH –COOH groups in the dehydration of D-glucose to 5-HMF. New dehydration tests were performed in mixtures of benzoic acid or derivatives with boric acid and choline chloride (molar ratio: 1/1/4) during 1 h at 90°C. Those benzoic acid derivatives are depicted in Figure 5. The choice of benzoic acid derivatives as model compounds to better understand the reaction is based on several elements. Firstly, benzoic acid derivatives are widely available at moderate price and show a large diversity of chemical structures. Secondly, compared to linear molecules possessing similar functions, they generally have higher degradation temperature and present a rigid structure which does not enable conformation changes (chemical functions on the benzene ring are always at the same distance).

**Figure 6:**
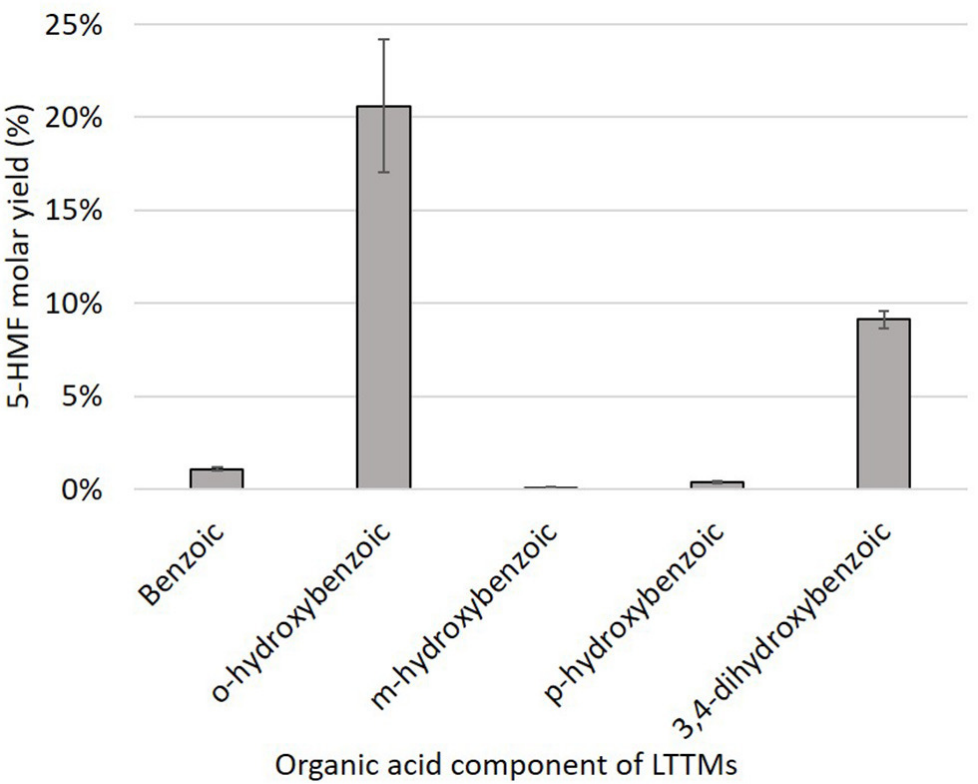
5-HMF yields obtained from D-glucose (0.56 mmol) in mixtures of choline chloride (10 mmol), benzoic acid derivatives (2.5 mmol), and boric acid (2.5 mmol) after 1 h at 90°C, 210 rpm.

While benzoic, *m*-hydroxybenzoic and *p*-hydroxybenzoic acids did not enable the production of more than 2% of 5-HMF (Figure 6), *o*-hydroxybenzoic acid rapidly converted D-glucose to 5-HMF (yield of 20.60 ± 3.59%), which confirmed the necessity of a –OH group nearby the carboxylic acid function. Again, humins rapidly appeared. Surprisingly, an intermediate 5-HMF yield of 9.11 ± 0.49% was achieved with 3,4-dihydroxybenzoic acid suggesting that acids with a diol moiety also enhance the reaction.

**Figure 7:**
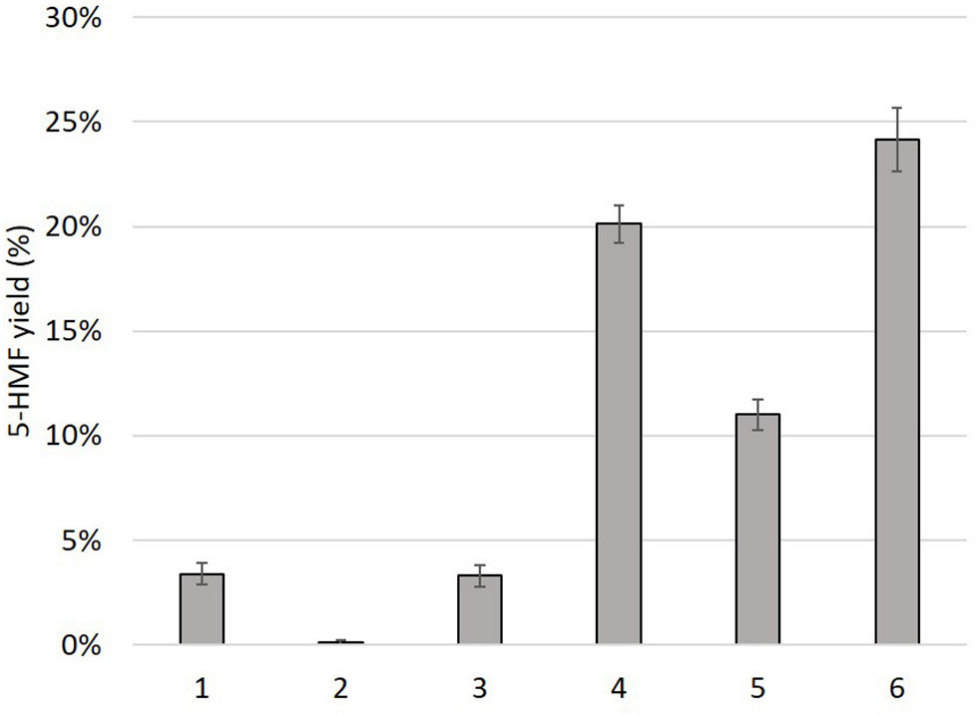
5-HMF yield (%) from D-glucose heated 1 h at 100°C in mixtures of choline chloride, boric acid, organic acids and/or hydroxyphenol. Each mixture contains water (10 wt%). 1: benzoic acid + 1,2-dihydroxybenzene, 2: phenol, 3: 1,2-dihydroxybenzene, 4: o-hydroxybenzoic acid + phenol, 5: o-hydroxybenzoic acid + 1,2-dihydroxybenzene, 6: o-hydroxybenzoic acid.

An additional test with a mixture of benzoic acid and 1,2-dihydroxybenzene led to a low amount of 5-HMF (Figure 7, result 1). The involvement of a vicinal diol or a -OH moiety nearby the acid function was further confirmed when 1,2-dihydroxybenzene (2.5 mmol) was added to the mixture containing o-hydroxybenzoic acid (Figure 7, result 5). The resulting 5-HMF yield decreased significantly suggesting that 1,2-dihydroxybenzene interfered with D-glucose conversion to 5-HMF, most probably because of its vicinal diol moiety. It is worth noting that the mixture of choline chloride, boric acid, and 1,2-dihydroxybenzene enabled the formation of 5-HMF without the addition of an organic acid (Figure 7, result 3), even at a lower extent.

The enhanced generation of 5-HMF and humins in the presence of boric acid and specific organic acids (α-hydroxyacids, *o*-hydroxybenzoic, and 3,4-dihydroxybenzoic) led us to consider the formation of tetrahydroxyborate esters (THBE) as a potential explanation of the catalytic mechanism. Boric acid and its corresponding tetrahydroxyborate anion (THB) are known to reversibly form borate esters with monosaccharides. More generally, they can form borate esters with diols (Pappin et al., 2012; Peters, 2014). Interestingly, boric acid and THB can also react with some organic acids with –OH group nearby the acid function. Oxalic, glycolic, tartaric, and lactic acids THBE have been observed as well as *o*-hydroxybenzoic acid THBE (Queen, 1977; Rebstöcková and Bartušek, 1977; Pizer and Selzer, 1984). THBE involving one THB and two molecules of lactic acid or two molecules of monosaccharides have also been described (Pizer and Selzer, 1984).

**Figure 8:**
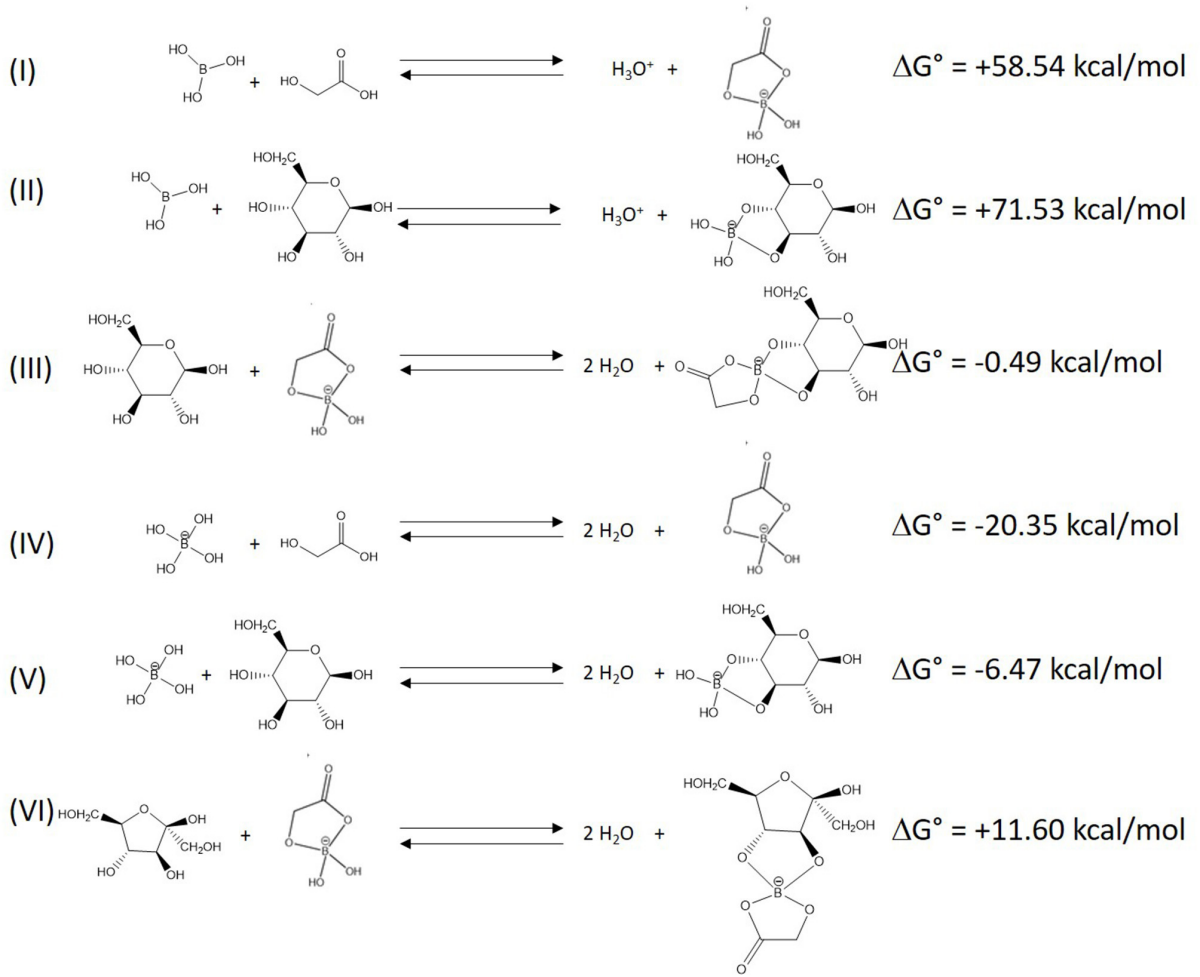
DFT (B3LYP/6-31G**)-calculated Gibbs free energies of reactions between boric acid, α-hydroxyacid, and/or a monosaccharide. In all cases, solvent effects have been taken into account through the use of the PCM scheme.

To get insights into the reaction mechanisms leading to the formation of 5-HMF from D-glucose, DFT calculations were performed and, in particular, the Gibbs free energies of selected reactions involving boric acid, α-hydroxyacid, and/or a monosaccharide were estimated (Figure 8). The reaction of boric acid with the α-hydroxyacid is a more favorable process (I) than that with glucose (II), suggesting that organic acid-THBEs are first formed and then react with the monosaccharide. When considering D-glucose, the latter reaction is exergonic (III) and produces water molecules, which can lead to the formation of THB from boric acid. As found for boric acid, our calculations suggest that THB will also preferentially associate with the α-hydroxyacid (IV) than with D-glucose (V). Organic acid-THBEs are definitely the chemical species that will react with monosaccharides; the reaction between boric acid and D-glucose followed by the association with an organic acid is very unlikely. This result is consistent with previous works that demonstrated that THBEs formed with carboxylic acids were more stable than THBEs formed with diols of D-glucose or 1,2-dihydroxybenzene (Babcock and Pizer, 1980). The lower pKa (9.3) of 1,2-dihydroxybenzene compared to D-glucose (>10) could consequently explain the inhibition presented in Figure 7 (result 5). Interestingly, the calculations show that the nature of the monosaccharide plays an important role on the thermodynamic character of the reactions with THBE: the chemical reaction with D-glucose is slightly exergonic (III) while it is clearly endergonic for D-fructose (VI). This different behavior originates from the ring constraints of the monosaccharide, as confirmed by the values of the distance between the oxygen atoms of the two hydroxyl functions before and after reaction with THBE. For an isolated D-fructose molecule, this distance amounts to 3.24 Å while it is much smaller for D-glucose (2.84 Å). In contrast, when bound to the THBE, the distance between the two oxygen atoms is now similar whatever the monosaccharide (2.43 and 2.39 Å for D-fructose and D-glucose, respectively) and significantly smaller than for the isolated monosaccharides. The larger distortion from equilibrium found for D-fructose is in full consistency with the increase of the Gibbs free energies.

The formation of D-fructose/organic acid-THBE being endergonic (VI), the assistance of D-fructose dehydration to 5-HMF by the organic acid-THBE seems unlikely. After the isomerization step, D-fructose would rather be released from the THBE. In fact, the rapid conversion of D-fructose to 5-HMF in mixtures without boric acid (see Table 2) suggests that the THBE is not required for the dehydration step. However, if D-fructose reacts with THBE, its conversion to glucose-THBE is energetically favored (ΔG° = −10.61 kcal/mol) which could explain the selectivity loss observed in the presence of boric acid (Table 2).

In the light of the discussed experiments and DFT calculations, THBE could catalyze 5-HMF formation in several ways:

A first possibility is the catalyzed production of 5-HMF by in situ generation of H_3_O^+^ resulting from the reaction between boric acid and the α-hydroxyacid. In this case, THBE would act similarly to boric acid by stabilizing the acyclic form of D-glucose and favor the isomerization step. Dehydration of D-fructose to 5-HMF would then be accelerated by the increased acidity of the medium.

For the second possibility, THBE could enhance isomerization further than boric acid. DFT calculations shows that the formation of organic acid-THBE and their reaction with D-glucose (+58.05 kcal/mol) is energetically favored compared to the reaction of boric acid with the monosaccharide (+71.53 kcal/mol).

To demonstrate the in situ formation of H_3_O^+^, LTTMs containing choline chloride, boric acid and an organic acid (4/1/1 mol) were prepared again as well as a mixture of choline chloride and boric acid (2/1). Small amounts of a solution of thymol blue (in ethanol) were added to each mixture to compare their acidity based on indicator dissociation. The absorbance of the indicator protonated form was measured at 548 nm. According to the organic acid, mixtures acidity increases in the following sequence: acetic <formic < no organic acid <maleic <glycolic ~ malic (Supplementary Figure 4 and Supplementary Table 1). While formic and glycolic acids possess a nearly similar pKa (around 3.8), their mixtures with choline chloride and boric acid showed completely different acidity, strongly supporting borate ester formation with H_3_O^+^ release. The mixture containing only choline chloride and boric acid was somehow more acidic than mixtures with formic or acetic acid. This can be understood observing reaction (I) in Figure 8. THB formation could be partially suppressed by the acidity provided with non-α-hydroxyacids.

The in situ formation of H_3_O^+^ explains why 5-HMF formation is enabled in the presence of 1,2-dihydroxybenzene and 3,4-dihydroxybenzoic acid. The low to intermediate 5-HMF yields result from a moderate THBE formation compared to α-hydroxyacid-THBE which are formed in greater amounts.

**Figure 9:**
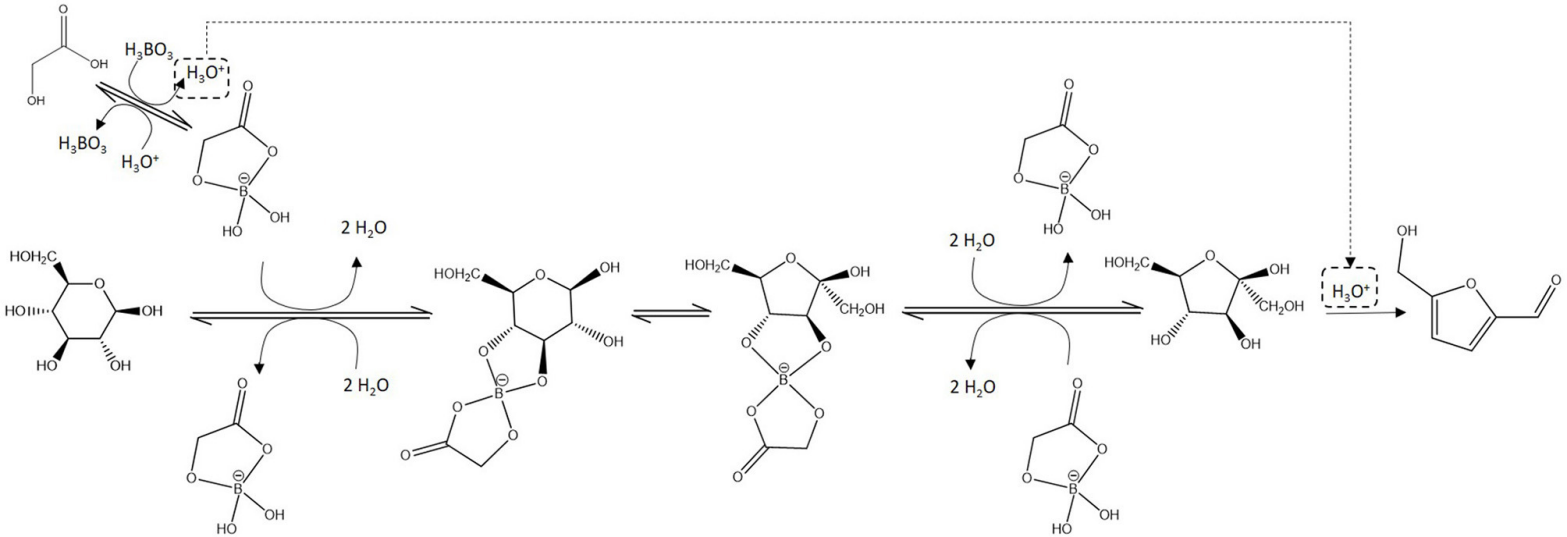
Mechanism for transformation of D-glucose into 5-HMF assisted by boric acid and α-hydroxyacids.

The proposed mechanism for 5-HMF formation assistance by THBE is depicted in Figure 9. α-hydroxyacids-THBE formation releases H_3_O^+^ and THBE react with D-glucose, favoring isomerization to D-fructose through a mechanism similar to the pathway proposed in the work of Stahlberg et al. (2011). Contrarily to metal catalysts (e.g., CrCl_3_, AlCl_3_, SnCl_4_, …), boric and boronic acids catalyze isomerization of aldoses to ketoses through an enediol intermediate rather than through a hydride shift. This was demonstrated in previous studies both experimentally with deuterium labeling experiments and theoretically at the DFT level (Stahlberg et al., 2011; Caes et al., 2013). We explained that ΔG° calculated for reaction of THB with glucose or fructose are different because of the ring constraints of the monosaccharides. When monosaccharide ring opens, these constraints are reduced which is why borate esters favor the acyclic form of monosaccharides (Stahlberg et al., 2011). It was also demonstrated that protonation of glucose O1, leading to the formation of a 1,2-enediol intermediate, is more favorable than in the absence of boric acid, which is probably because it is facilitated by the negatively charged borate (Stahlberg et al., 2011). After additional proton transfer, the enediol intermediate can be converted to fructose.

The catalysis with α-hydroxyacids-THBE is likely to proceed in a similar way even though two differences could occur compared to boric acid:

- The formation of organic acid-THBE and their reaction with D-glucose is energetically favored compared to the reaction of boric acid with the monosaccharide.
- In slightly acidic conditions, THBE, bearing only two available B-OH moieties, could limit the extent of polymerization reactions compared to boric acid or tetrahydroxyborate which possess three and four available B-OH moieties, respectively. While rarely addressed in the context of furan compounds synthesis, polymerization through coordination of several chemical species (glucose, 5-HMF) to a same catalyst has been experimentally suggested, specifically for vanadium trichloride (Li et al., 2014). This possibility is further discussed in part 3.4.

Free cyclic D-fructose being energetically favored compared to D-fructose-THBE, D-fructose could be released from THBE and initiate dehydration to 5-HMF catalyzed by H_3_O^+^. The proposed mechanism implies an important role of water for reversible hydrolysis of THBE, which may seem inconsistent with the initially anhydrous composition of the reaction medium. However, it is expected that the THBE formation releases a significant amount of water as proposed in Figure 9.

**Figure 10:**
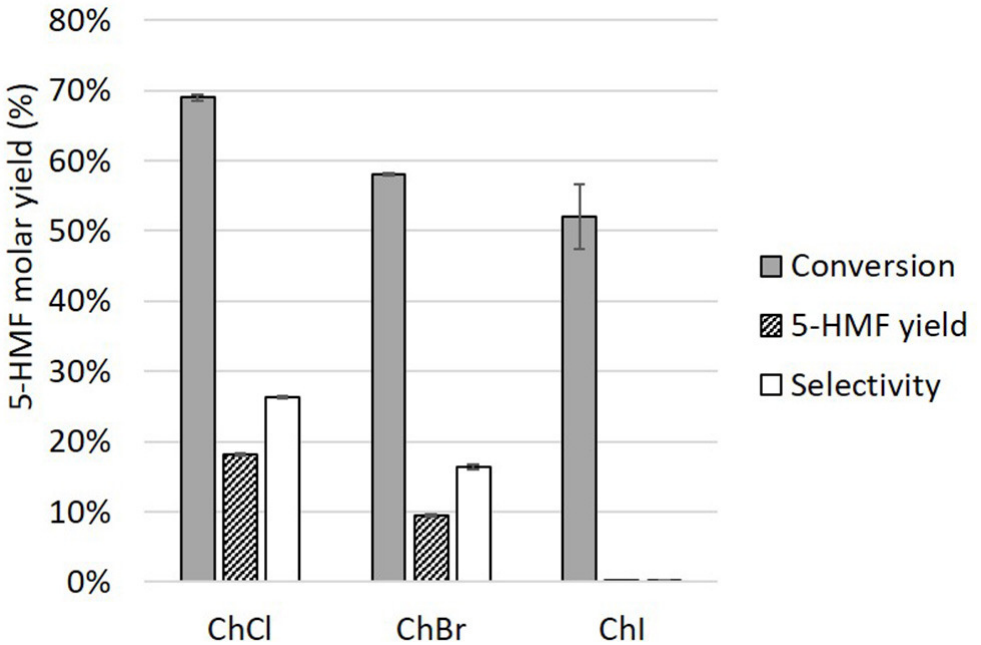
Comparison of choline chloride (ChCl), choline bromide (ChBr), and choline iodide (ChI) in mixture with boric and glycolic acids for the dehydration of D-glucose to 5-HMF (90°C, 210 rpm, 1 h).

After determining the role of organic and boric acids, it is important to understand the function of choline chloride. While the role of the choline cation is mainly to ensure the formation of a melted mixture at moderate temperature, halide anions are known to strongly affect monosaccharides dehydration. Different choline halides were used in mixture with boric and glycolic acids (4/1/1 mol) to precise the anion importance. The results (Figure 10) suggest that chloride is the most effective anion for the catalysis of D-glucose conversion to 5-HMF followed by bromide. The amounts of humins after the reaction were 6.60 ± 0.32% and 0.61 ± 0.20% for choline chloride, and choline bromide, respectively. Choline iodide led to negligible amounts of humins.

These results follow the same trend as highlighted in the work of Mellmer et al. (2019). The author studied D-fructose dehydration in γ-valerolactone with Bronsted acid and different salts. They demonstrated that chloride alone could not catalyze D-fructose, but greatly enhanced the dehydration reaction combined to an acid catalyst. The highly localized charge on chloride anions allows them to stabilize carbocations which are dehydration intermediate as well as their deprotonation transition state. Iodide being a strong reducing agent was likely oxidized to iodine which limited its efficiency. A fast color shift from transparent to yellow-brown was indeed observed in the LTTM during the test, which could be attributed to dipolar interactions between iodine and oxygen. Choline fluoride was not investigated since fluoride, being a weak base, could allow the formation of hydrofluoric acid (pKa = 3.2). Isolating the effect of fluoride anions would therefore not be possible.

1D ^1^H and 2D ^1^H-^13^C HSQC NMR analyses were performed on a mixture of choline chloride, oxalic and boric acids (4/1/1 mol) after 1 h of treatment at 90°C to confirm the formation of 5-HMF as well as to determine if LTTMs were stable in the tested conditions (Supplementary Figures 5–7). The presence of 5-HMF was confirmed by ^1^H-^13^C correlation signals at (9.3, 180.6) ppm for the aldehyde function, at (7.4, 126.9) and (6.6, 110.5) ppm for the furan ring C-H and at (4.6, 55.9) for CH_2_ near the –OH moiety. Large signals at (5.1, 92.0) and (4.6, 95.8) ppm are attributed to α-D-glucopyranose and β-D-glucopyranose, respectively. No signal corresponding to levulinic (2–3, 25–40 ppm) or formic (8.5, 174) ppm acid was observed. The stability of LTTMs composed of choline chloride and organic acids has been questioned since esterification reactions were reported (Rodriguez Rodriguez et al., 2019). Rodriguez Rodriguez et al. (2019) observed the appearance of NMR 1D ^1^H signals near 3.25, 3.8, and 4.7 ppm corresponding to choline ester formation in a mixture of choline chloride and oxalic acid. Regarding the choline chloride, oxalic acid, boric acid mixture, a small signal is present near 3.12 ppm but no other signal was observed (Supplementary Figure 5). The esterification of choline seems therefore limited or absent which is surprising considering the known ability of boric acid to catalyze esterification. This could be specific to the presence of boric acid in equimolar quantity with the organic acid with THBE formation preventing the organic acid from esterification. The presence of D-glucose is also of importance because the monosaccharide, with vicinal diol moieties, will likely react in priority comparing to the simple alcohol function of choline.

It was suggested in part 3.1 that esterification of glucose with organic acid could be a main side reaction in mixture of choline chloride and organic acid to explain conversion trends in Figure 2. A ΔG° of +5.07 kcal/mol was calculated for the esterification of glucose with glycolic acid at glucose O1 position. This value suggests that esterification is indeed possible from an energetic point of view. However, the presence of boric acid reduces aldoses conversion (glucose, xylose, arabinose) and promotes furan derivatives in Table 2. Again, this could be explained if boric acid limits the extent of esterification by reacting with the organic acid.

### Comparison of Monosaccharide Dehydration in LTTMs

**Figure 11:**
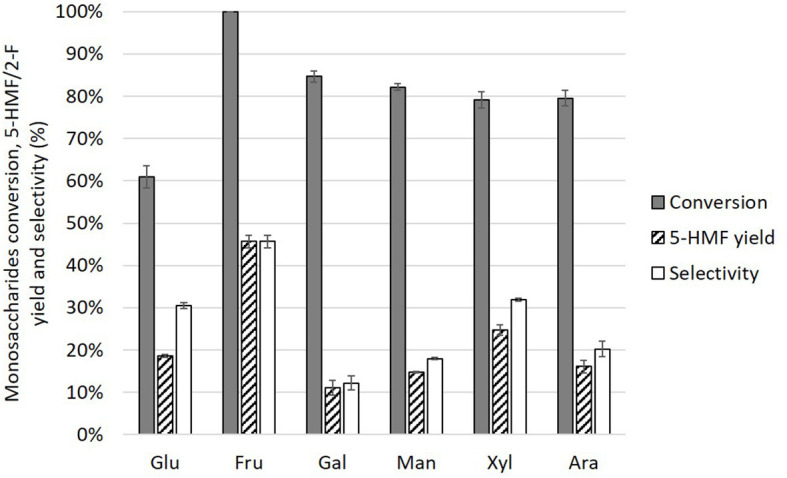
Conversion of monosaccharides (0.56 mmol) and 5-HMF/2-F yield/selectivity in a mixture of choline chloride (10 mmol), boric acid (2.5 mmol), and glycolic acid (2.5 mmol) after 1 h at 90°C.

The synergy between boric acid and the α-hydroxyacid was evaluated for other monosaccharides: D-fructose, D-mannose, D-galactose, D-xylose, and D-arabinose. Monosaccharide conversion, 5-HMF/2-F yields (for hexoses and pentoses, respectively) and selectivity after a treatment of 1 h in a mixture of choline chloride, glycolic acid, and boric acid (molar ratio: 4/1/1) are depicted in Figure 11. Compared to the preliminary test with maleic and boric acid, the dehydration selectivity of all aldoses to furan derivatives is improved. For D-fructose however, the selectivity for 5-HMF is slightly reduced since isomerization to D-glucose enables the formation of side-products (e.g., humins). Humins were observed after the treatment of each monosaccharide (Supplementary Figure 8).

Compared to D-glucose, 5-HMF synthesis from D-mannose, and D-galactose were less selective. This was tentatively explained in the work of Lukamto et al. (2013). Complexation of boric/boronic acids with cis vicinal diol is favored compared to complexation with trans vicinal diol. Observing hydroxyl positions on C2, C3, and C4 of aldoses pyranose ring, it can be concluded that D-glucose possesses two trans vicinal diol moieties while mannose and galactose only possess one. Since complexation of trans vicinal diol would induce a distortion of the pyranose ring, the opening of the aldose cyclic form could be favored, which is a key step of the isomerization process. The lower selectivity achieved with D-galactose can also be rationalized considering its corresponding ketose, D-tagatose, which is more reactive than D-fructose and a less selective substrate for 5-HMF synthesis (Van Putten et al., 2017).

### Humins Characterization

Humins have been described as polymers resulting from aldol addition/condensation reactions of 5-HMF and possibly monosaccharides. 2,5-dioxo-6-hydroxy-hexanal, a rehydration product of 5-HMF, has been proposed as a key precursor of humins. In this work, humins formation from hexoses and pentoses was observed in all mixtures containing boric acid and was particularly pronounced in mixtures also containing α-hydroxyacids. A humin mass yield of 21.13 ± 0.51% was obtained from D-glucose after a 2 h treatment in a mixture of choline chloride, boric acid, and glycolic acid (4/1/1 mol). To better understand how humins are generated in LTTMs, further experiments were conducted. When D-glucose was replaced by 5-HMF in the same LTTM, a humins mass yield of only 1.23 ± 0.11% was achieved after 2 h suggesting a major role of D-glucose (Supplementary Figure 9). Additional tests with LTTMs containing only boric acid and choline chloride or only glycolic acid and choline chloride confirmed that D-glucose, boric acid and the α-hydroxyacid were required to produce large amounts of humins. Interestingly, when equimolar quantities of D-glucose and 5-HMF were used together as the reaction substrate in the mixture of choline chloride, boric acid, and glycolic acid, the humins yield was twice higher than the expected combined yields from individual substrate (Supplementary Figure 10).

Assays being performed under air, oxidation reactions cannot be excluded. To detect any potential effect on humins formation, glucose dehydration in a choline chloride, glycolic acid, boric acid mixture (4/1/1 mol) was performed under nitrogen or air. After 1 h at 90°C, a humins mass yield of 10.65 ± 0.96% was achieved under nitrogen, which is similar to the humins yield obtained under air (10.33 ± 0.90%). The involvement of oxidation seems therefore limited in humins formation in the tested conditions.

To get further insight into humins formation mechanism, infrared spectra of humins were recorded (Supplementary Figure 11). In hexose humins spectra, double peaks are observed near 1,700 and 1,670 cm^−1^ corresponding to conjugated aldehyde or conjugated ketone stretching. A broader peak is found near 1,590 cm^−1^. Bands between 800 and 740 cm^−1^ correspond to out of plane =CH bending. Such bands have been observed in humins incorporing 5-HMF and are thought to originate from the furan ring C-H bending. The peak at 795 cm^−1^ is followed by a small shoulder near 760 cm^−1^ which suggests that out of plane =CH bending has several possible origins besides 5-HMF. The presence of the furan ring is nevertheless supported by a small =CH stretching band near 3,110 cm^−1^. IR spectra indicate that humins likely contain C=C-CO in their structure and that 5-HMF participates to the polymerization reaction.

Humins generated from pentoses show some differences: only one band is present near 1,700–1,670 cm^−1^ and two well-defined =CH bending bands are present at 790 and 743 cm^−1^. A broad band is still observed near 1,600 cm^−1^ for D-xylose and D-arabinose humins.

Regarding =CH bending, the band at 743 cm^−1^ in xylose and arabinose humins can clearly be attributed to =CH bending of 2-furfural ring as observed from the comparison with 2-furfural infrared spectrum (Supplementary Figure 12) which confirm the incorporation of the furan ring. The peak at 790 cm^−1^ could again be attributed to =CH bending from tri-substituted alkenes generated by aldol addition/condensation. After infrared analysis of pure 5-HMF (Supplementary Figure 13), a =CH bending peak was observed near 780 cm^−1^ which could explain the bands superposition observed for hexoses humins in this region of the spectra.

2D HSQC ^1^H ^13^C NMR provided additional elements to understand humins formation. Humins formed from D-fructose in a mixture of choline chloride, boric acid, and glycolic acid (4:1:1 mol) at 90°C during 60 min were collected and thoroughly washed with water then dried before dissolution in d6-DMSO for 2D NMR analyses (Supplementary Figures 14, 15). It appears that numerous hydroxylated carbons (around 3–4; 60–70 ppm) are still present in the structure likely originating from monosaccharide incorporation. 5-HMF furanic structure is also likely present according to signals near (6.47; 112.46 ppm) and (7.42; 124.76) as well as a signal at (4.66, 63.57). A signal at (9.49; 177.99 ppm) confirms that aldehyde moieties remain present in humins, ensuring the possibility of further polymerization by adol addition/condensation reactions. The signal at (8.08; 130.09 ppm) could originate from aldol addition/condensation between monosaccharide molecules forming a heavily conjugated structure.

Finally, conversion of D-glucose into 5-hydroxymethylfurfural was performed again in a mixture of choline chloride, boric acid, and glycolic acid (molar ratio: 4/1/1) during 1 h at 90°C. In order to create a competition between aldehydes for aldol condensation reactions, a large amount of 2-furfural (2.6 mmol for 0.56 mmol of D-glucose) was added to the mixture. It was expected that the introduction of 2-furfural in the reaction medium should not inhibit humins formation but could however partially replace 5-HMF in the polymer structure. Not only was 2-furfural incorporated in D-glucose humins as revealed by the presence of a new infrared band near 750 cm^−1^ (Supplementary Figure 16) for the recovered and washed insoluble, but the selectivity of D-glucose transformation into 5-HMF was improved from 30.48 ± 0.76% to 37.86 ± 1.89% as expected (Supplementary Figure 17). D-glucose conversion remained exactly the same as without added 2-furfural but 5-HMF yield was slightly higher. 5-HMF incorporation in humins was limited thanks to the higher concentration of 2-furfural. Generated humins reached 23.63 ± 0.75% of D-glucose initial mass which is twice as much as humins produced without added 2-furfural. Two bands at 1,700 and 1,670 cm^−1^ were still observed which confirms that their appearance is dependent of the initial substrate rather than the furanic derivative structure.

From all the gathered data, humins formation is likely favored by the acidity of the medium as well as by boric acid. In a mixture of choline chloride (10 mmol) and boric acid (2.5 mmol) containing hydrochloric acid (2.5 mmol), no glucose remained after 1 h and a 5-HMF yield of 16.76 ± 0.44% was achieved. Humins yield reached as high as 34.66 ± 0.10% of D-glucose initial mass. In the absence of α-hydroxyacid and considering the strong acidity provided by HCl, it is likely that THB is absent of the mixture, leaving boric acid as the only present boron specie. In a similar fashion to metallic catalysts (e.g., CrCl_3_, AlCl_3_,…) (Li et al., 2014), boric acid likely enables polymerization side-reactions by simultaneously bonding to several reactive species (e.g., two monosaccharides molecules, 5-HMF, 2-F,…).

We suspect that the number of available B-OH groups affects the reaction selectivity. The proportion of THBE relative to the initial boric acid content in the tested LTTMs is not known. THBE, possessing only two available B-OH moieties could limit the extent of polymerization reactions compared to boric acid or tetrahydroxyborate which possess three and four available B-OH moieties respectively. Moreover, the dehydration rate is likely faster than the isomerization rate since no fructose could be observed during the reaction.

The reaction of boric acid with diols (e.g., ethylene glycol, 1,2-dihydroxybenzene) does not likely enhance the selectivity like α-hydroxyacids or *o*-hydroxybenzoic acid do. We tentatively explain this with the stability of the formed borate complexes. α-hydroxyacids-THBE are more stable than diol-THBE in acidic conditions. The fraction of bound α-hydroxyacid or bound diol is strongly pH dependent. A comparison of borate esters formation from α-hydroxyacid or from ethylene glycol in water is depicted in the work of Peters (2014). A diol bound to boric acid could easily be replaced by a monosaccharide which possesses a close affinity. In slightly acidic conditions, diol would therefore not limit polymerization as α-hydroxyacids would. THBE catalysis is not straightforward: a precise ligand and a fine tuning of the acidity are likely required to achieve selective furan derivatives synthesis. These elements offer interesting perspectives for the design of new heterogeneous catalysts and can be transposed to catalysis by transition metals which is also impacted by ligand type and acidity conditions.

Further investigations should be conducted on THBE catalysis of monosaccharides dehydration. More specifically, mixtures of choline chloride, boric acid, and α-hydroxyacid could be partially neutralized in order to promote THBE as well as decreasing the acidity of the medium in order to reduce humins formation.

## Conclusion

Boric acid enables isomerization of aldoses to ketoses to a limited extent in choline chloride/organic acid based LTTMs (e.g., 5% 5-HMF yield and 23% glucose conversion after 1 h at 90°C with maleic acid). Combined with α-hydroxyacids however, the reaction is faster and more selective (e.g., 19% 5-HMF yield and 61% glucose conversion after 1 h at 90°C). The synergy between α-hydroxyacids and boric acid is explained by formation of tetrahydroxyborate esters. Compared to boric acid, THBE reaction with glucose is energetically favored. THBE formation is associated with H_3_O^+^ release in the medium increasing its acidity.

During experimentation with α-hydroxyacids, humins are generated as the main side-product. Those humins originate from monosaccharide and furanic derivatives polymerization. Humins formation is likely favored by the high acidity produced from THBE formation and possibly by residual boric acid.

We suggest that furanic derivatives synthesis could be promoted by a partial neutralization of the medium in order to drive THBE formation and limit the proportion of boric acid as well as reducing acidity to better balance isomerization and dehydration rates. Consequently, boric acid potential as a catalyst for furan derivatives synthesis has not been fully explored yet. THBE catalysis should be further investigated because it could improve performances of cheap and abundant catalyst (boric acid and organic acids).

## Data Availability Statement

All datasets presented in this study are included in the article/Supplementary Material.

## Author Contributions

TI: main dehydration experiments, mechanistic investigations, and manuscript writing. VL: density functional theory calculations and manuscript writing/correction. GD: competition experiments. LB: mechanistic investigations and manuscript correction. RL: density functional theory calculations and manuscript correction. AR: manuscript writing and correction. All authors contributed to the article and approved the submitted version.

## Conflict of Interest

The authors declare that the research was conducted in the absence of any commercial or financial relationships that could be construed as a potential conflict of interest.
