# Supplementary material for: Monosaccharides Dehydration Assisted by Formation of Borate Esters of α-Hydroxyacids in Choline Chloride-Based Low Melting Mixtures
Source: Front Chem. 2020 Jul 7;8:569. doi: 10.3389/fchem.2020.00569 (PMC7358950; doi:10.3389/fchem.2020.00569)
Supplement: Supplementary file 1 [file Data_Sheet_1.PDF]

Supplementary Material

1 Supplementary Figures

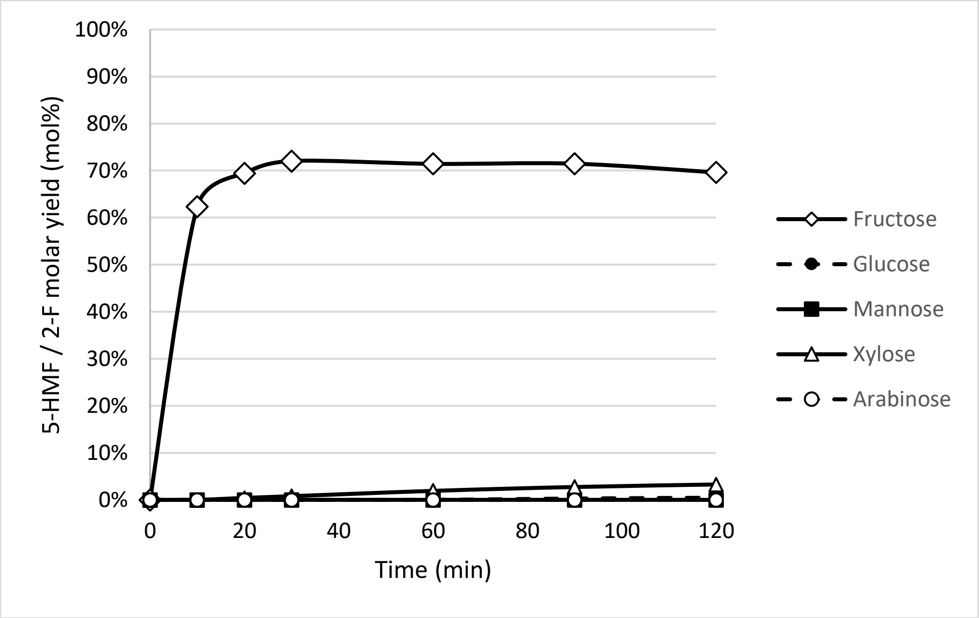

Supplementary Figure 1. Evolution of 5-HMF and 2-F molar yields (mol%), for hexoses (0.56 mmol) and pentoses (0.67 mmol) respectively, in a mixture of choline chloride (10 mmol) and maleic acid (5 mmol) at 90°C.

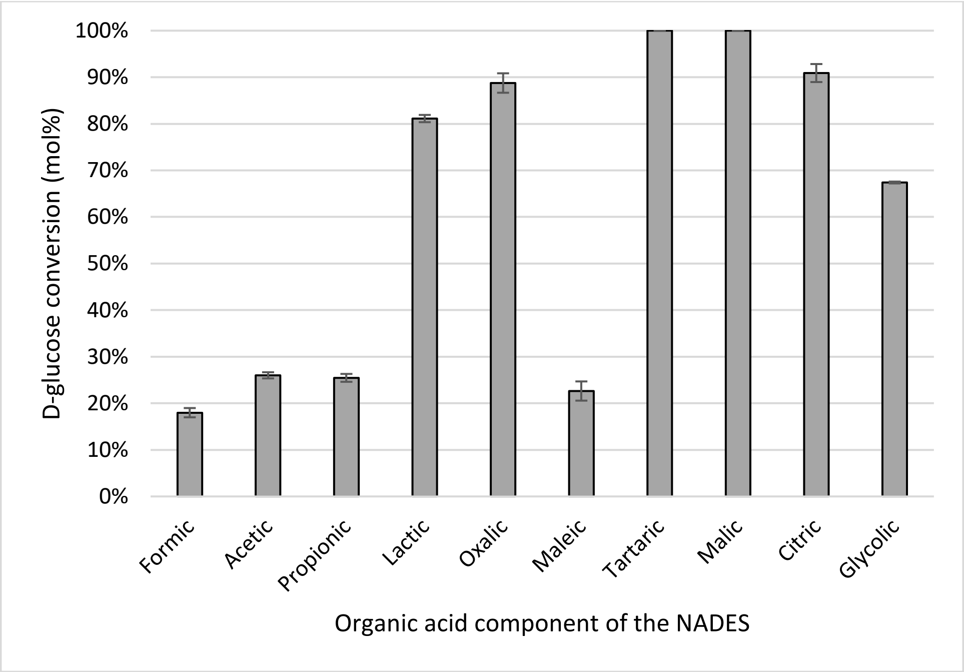

Supplementary Figure 2. D-glucose conversion (0.56 mmol) in mixtures of choline chloride (10 mmol), organic acid (2.5 mmol) and boric acid (2.5 mmol) after 1 h at 90°C, 210 rpm.

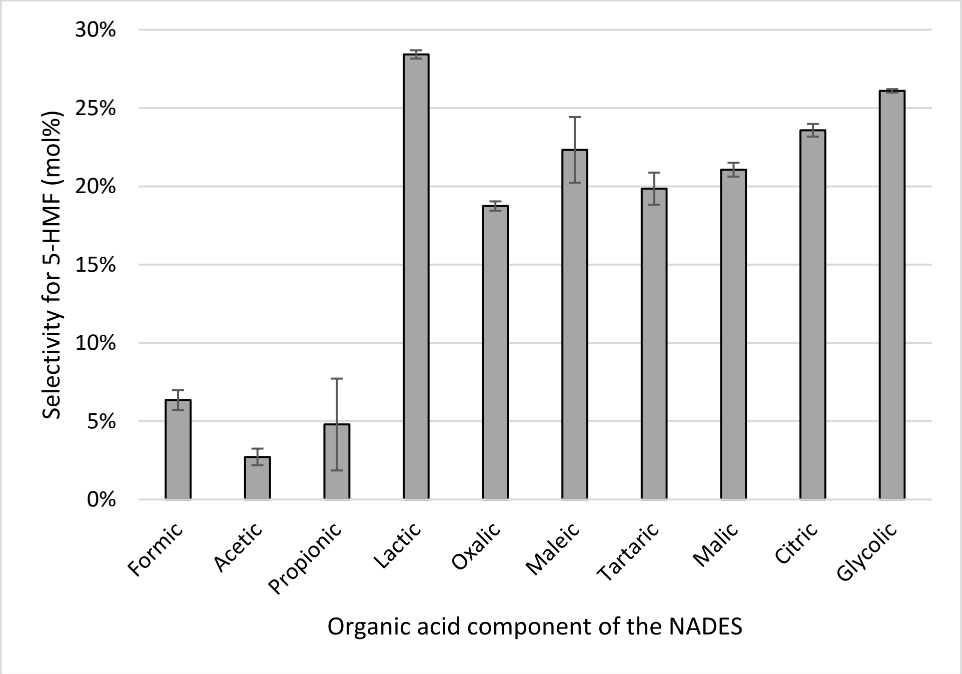

Supplementary Figure 3. Selectivity of D-glucose (0.56 mmol) transformation to 5-HMF in mixtures of choline chloride (10 mmol), organic acid (2.5 mmol) and boric acid (2.5 mmol) after 1 h at 90°C, 210 rpm.

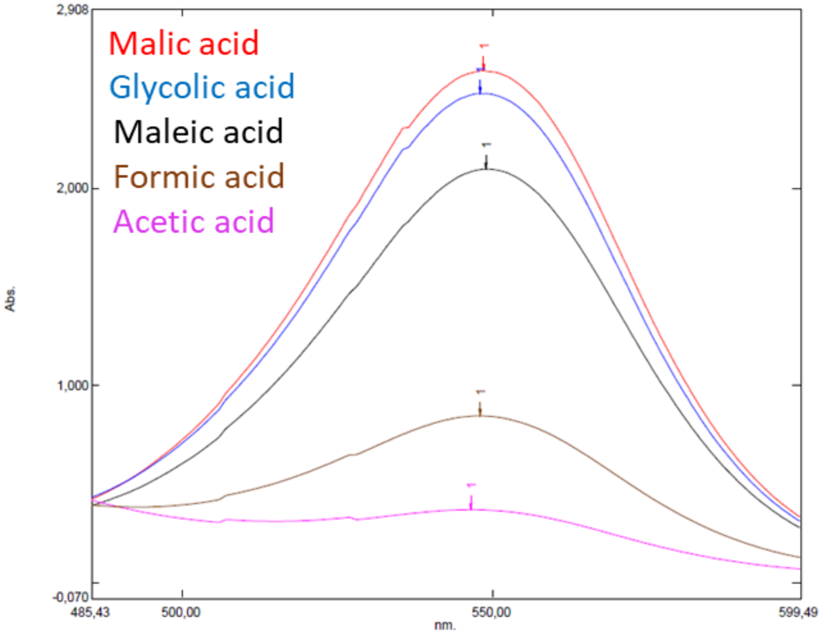

Supplementary Figure 4. Absorbance (from 485 to 600 nm) of different mixtures of choline chloride (20 mmol), boric acid (5 mmol) and organic acid (5 mmol) containing 13 wt% water and 50 µL of thymol blue in ethanol (0.05 g/10 ml).

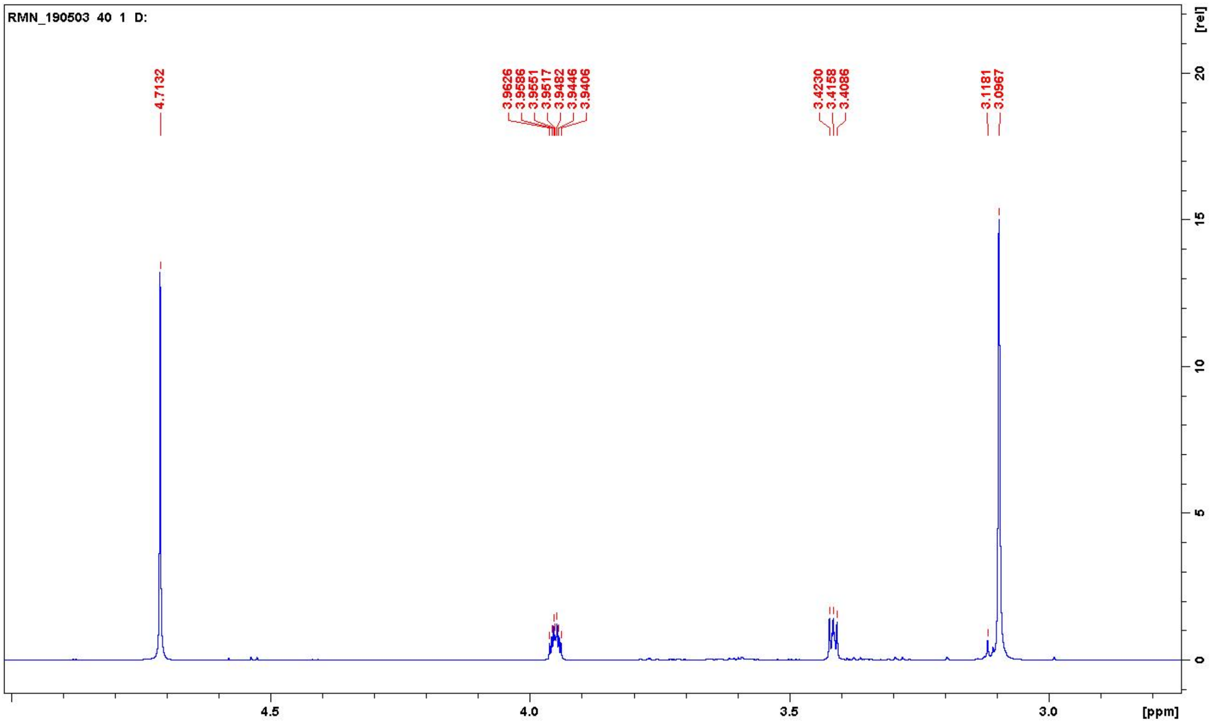

Supplementary Figure 5. 1D 1H spectrum of a mixture of choline chloride, boric acid and oxalic acid (4/1/1 mol) after a 1h treatment of D-glucose at 90°C. The sample was dissolved in D2O before analysis.

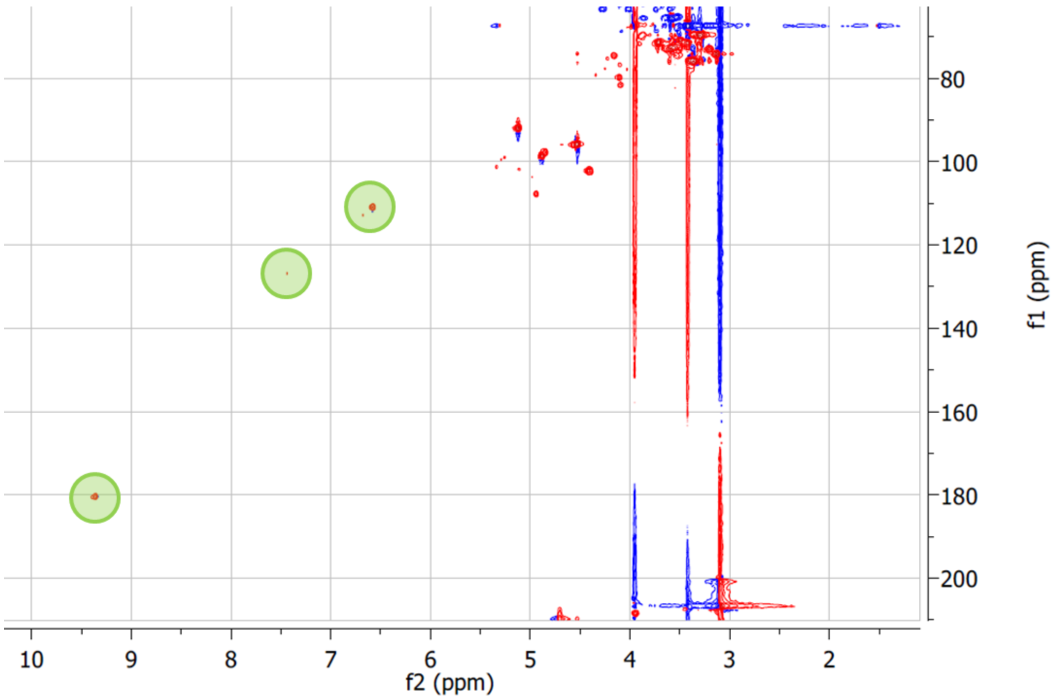

Supplementary Figure 6. HSQC NMR spectrum after treatment of D-glucose 1 hour in a mixture of choline chloride, oxalic acid and boric acid (signals attributed to 5-HMF are highlighted in green)

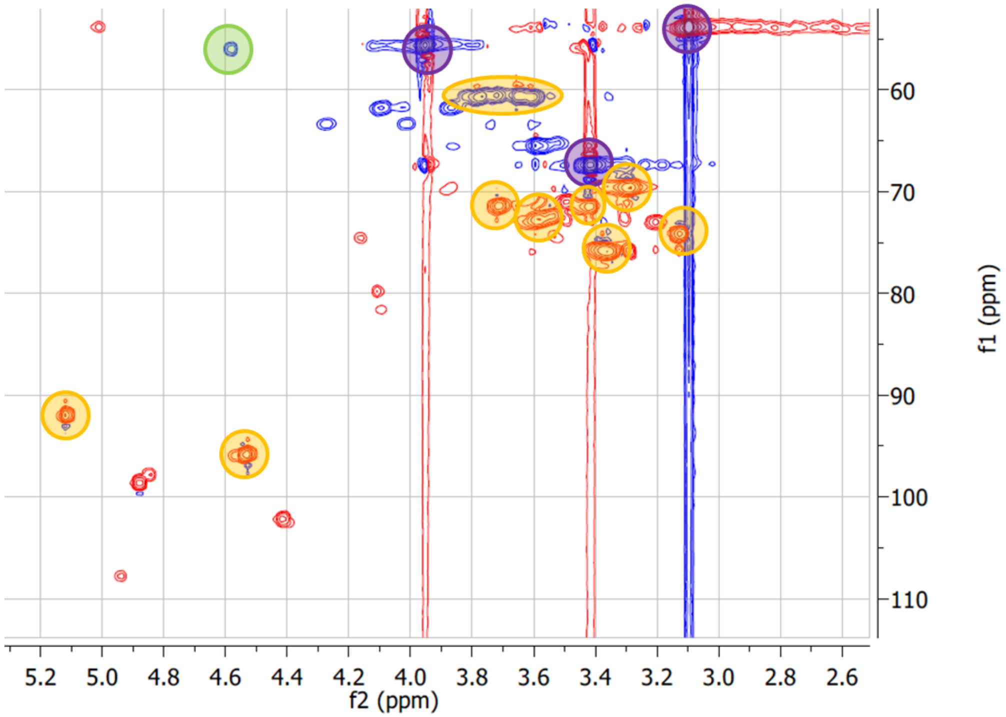

Supplementary Figure 7. HSQC NMR spectrum after treatment of D-glucose 1 hour in a mixture of choline chloride, oxalic acid and boric acid (Signals were attributed to molecules as follows: Green: 5-HMF, Purple: choline chloride, Yellow: D-glucose)

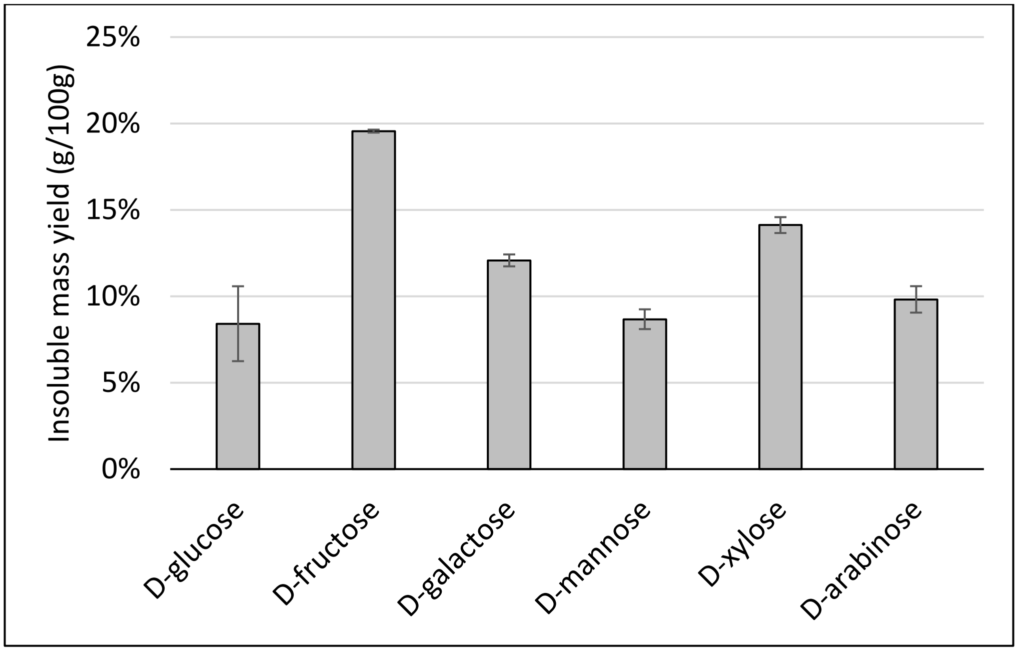

Supplementary Figure 8. Humins mass yields generated from monosaccharides in a mixture of choline chloride, boric acid and glycolic acid after one hour at 90°C.

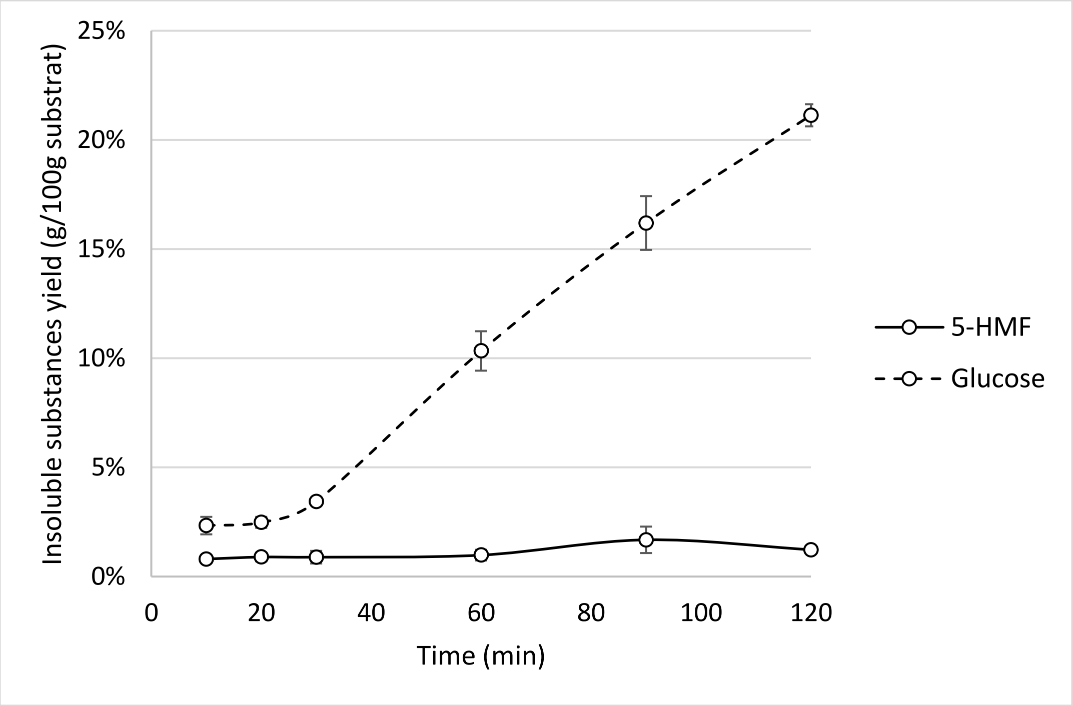

Supplementary Figure 9. Mass yields of generated humins during treatment of D-glucose (0.00056 mol) or 5-HMF (0.00056 mol) in a mixture of choline chloride (0.0100 mol), glycolic acid (0.0025 mol) and boric acid (0.0025 mol) after one hour at 90°C, 210 rpm.

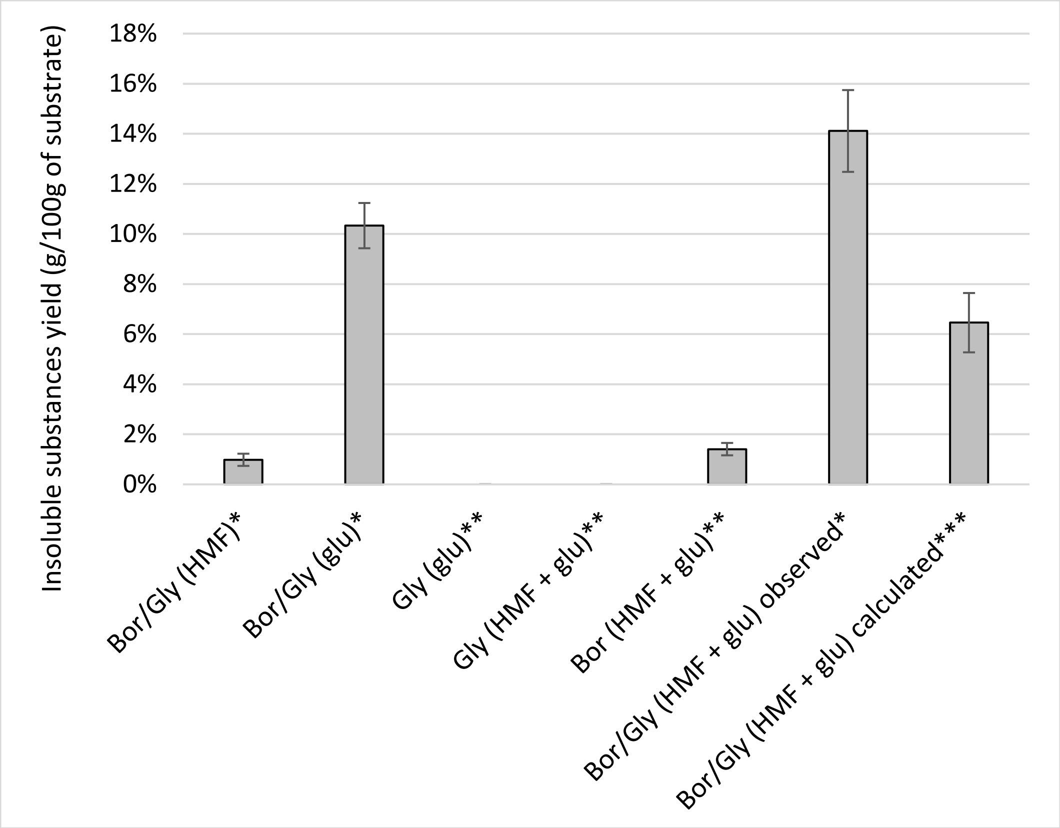

Supplementary Figure 10. Mass yields of generated humins during treatment of D-glucose (0.00056 mol), 5-HMF (0.00056 mol) or a mixture of both substrates (0.00028 mol + 0.00028 mol) in mixtures of choline chloride (0.0100 mol), glycolic acid and/or boric acid after one hour at 90°C, 210 rpm. *Those LTTMs contain 0.0025 mol of glycolic acid and 0.0025 mol of boric acid. **Those LTTMs contain 0.0050 mol of glycolic acid or boric acid. ***The calculated yield is obtained considering that the mixture of 5-HMF and D-glucose generates humins in a similar way to separated substrates.

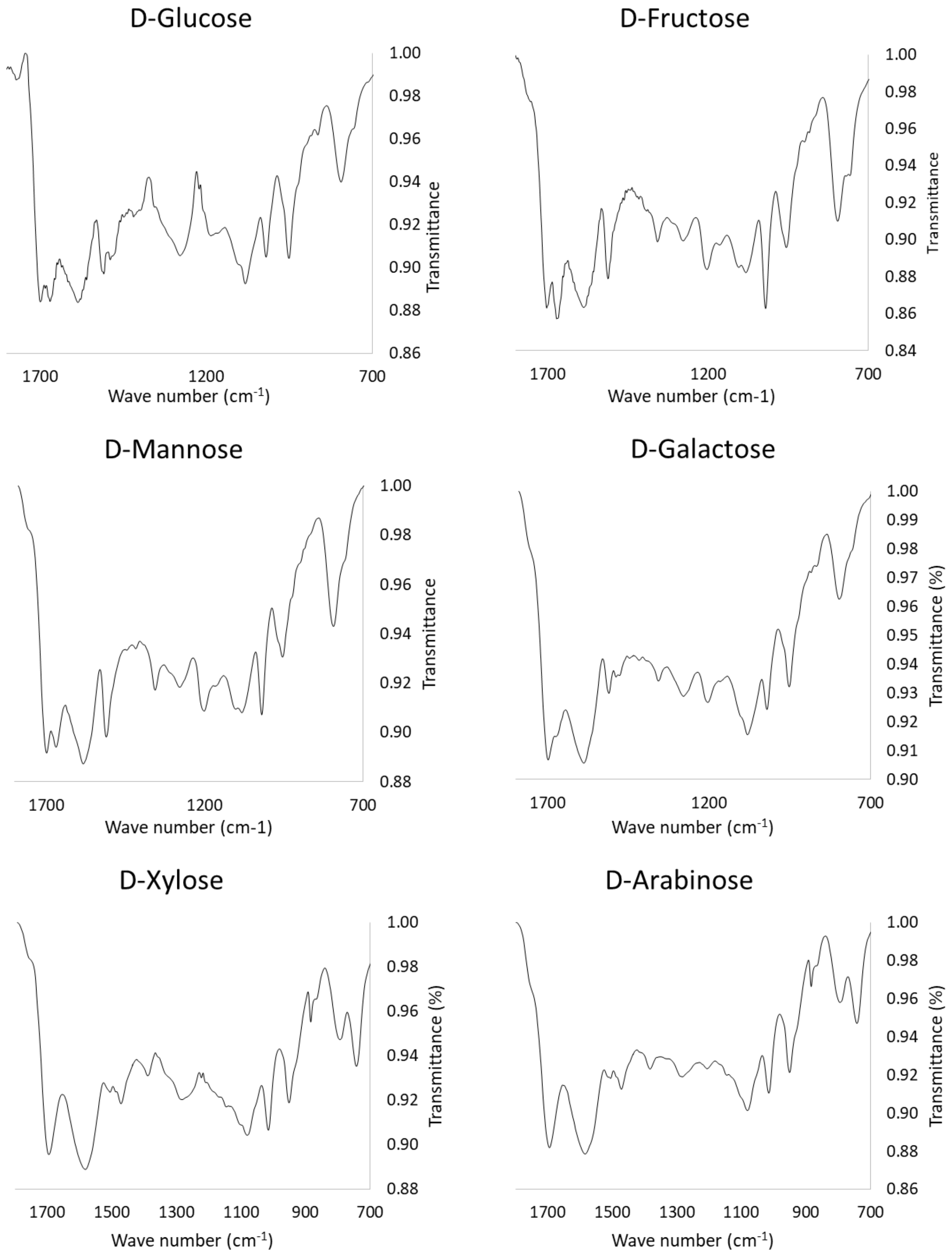

Supplementary Figure 11. Infrared spectra of humins

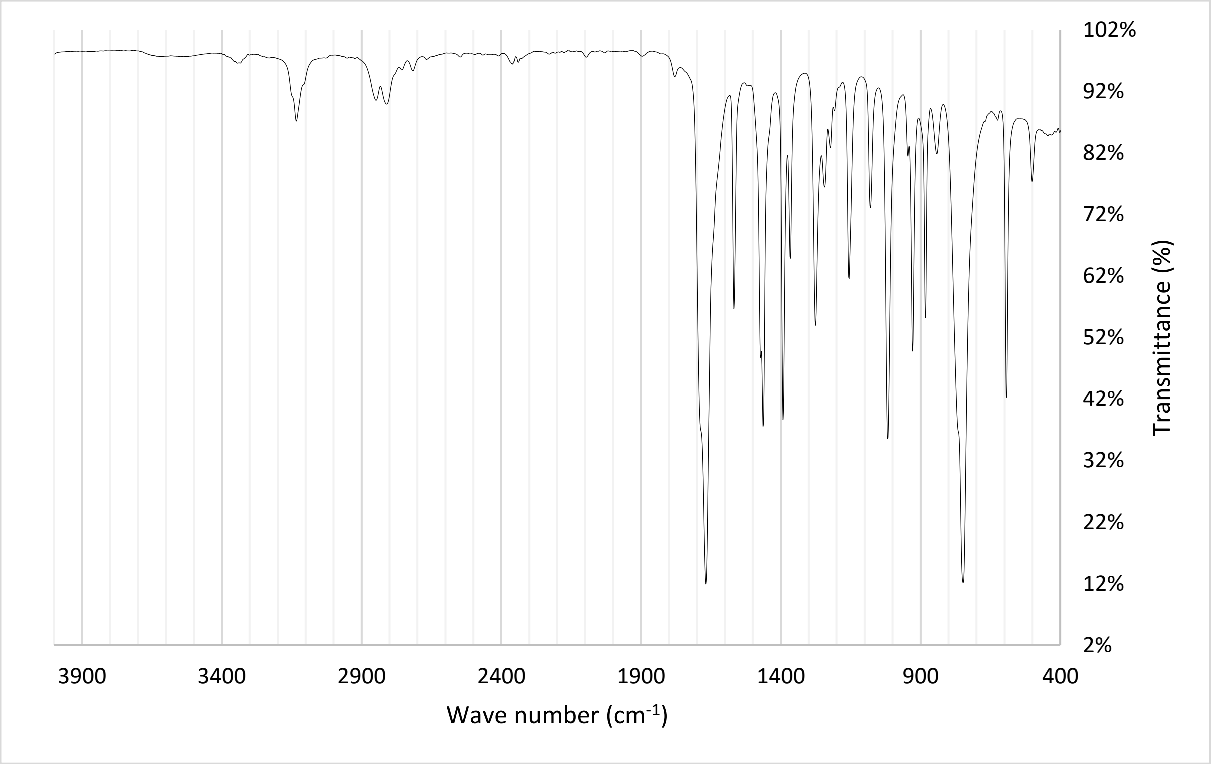

Supplementary Figure 12. IR spectrum of 2-furfural

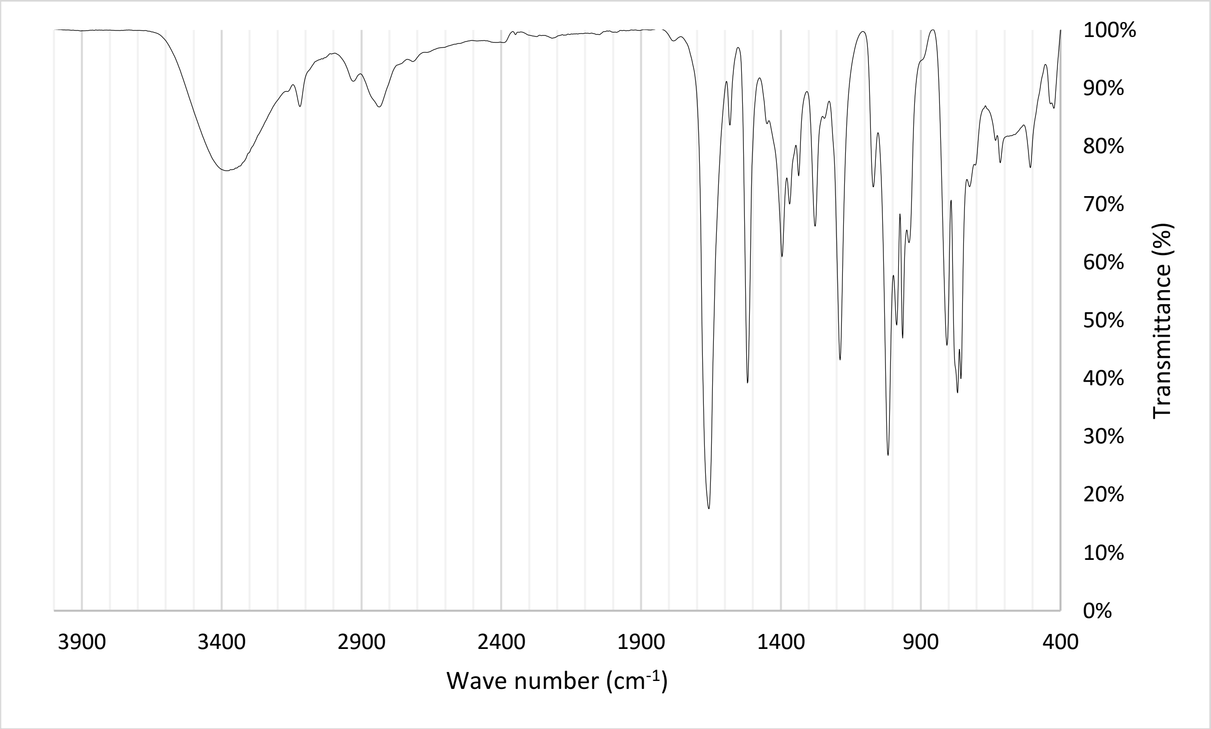

Supplementary Figure 13. IR spectrum of 5-HMF

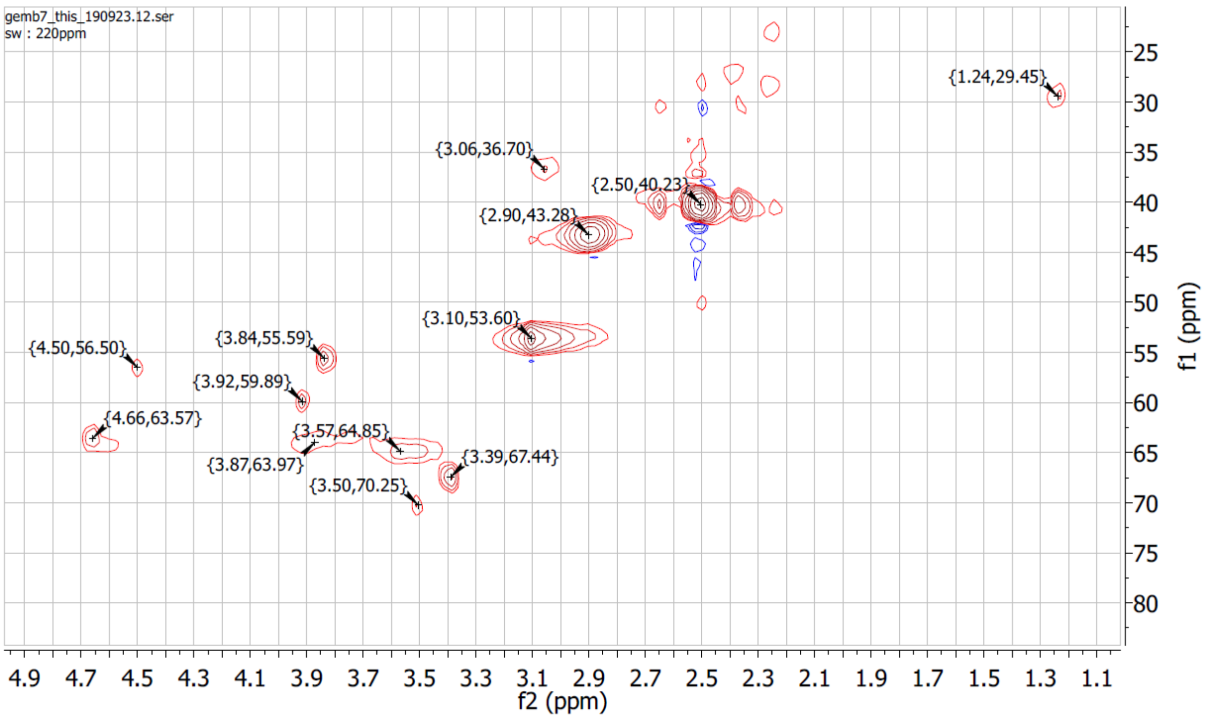

Supplementary Figure 14. 2D HSQC NMR spectrum of D-fructose humins

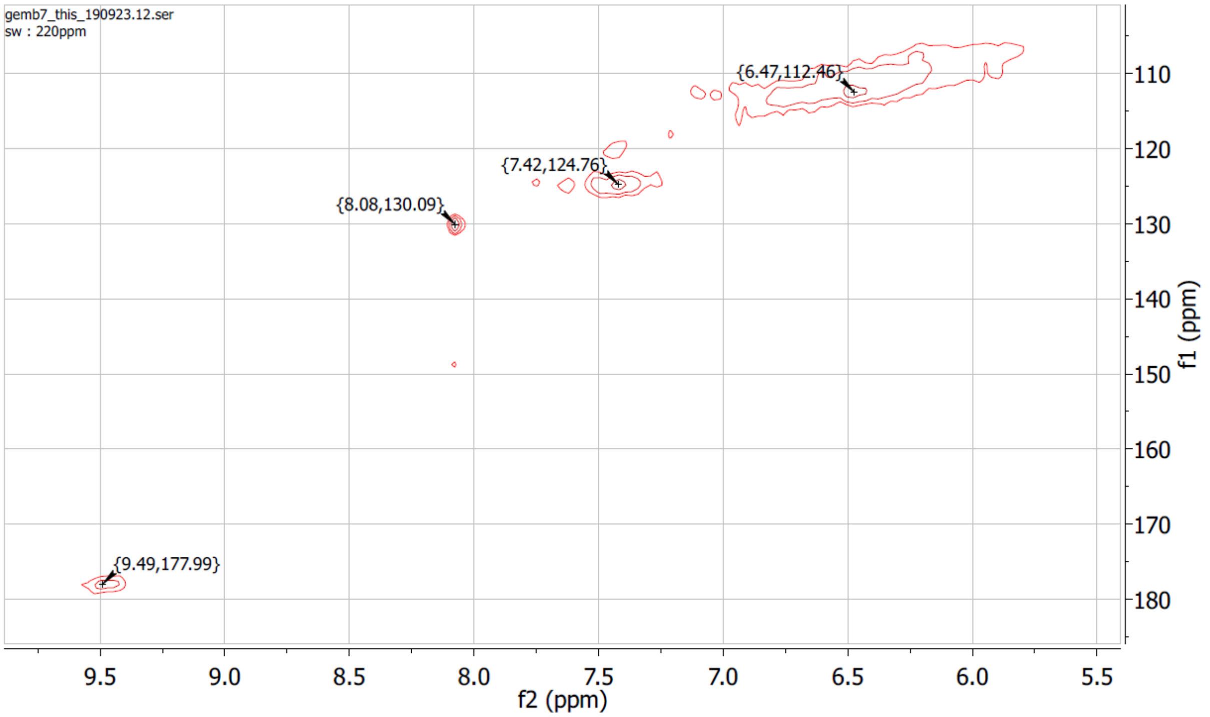

Supplementary Figure 15. 2D HSQC NMR spectrum of D-fructose humins

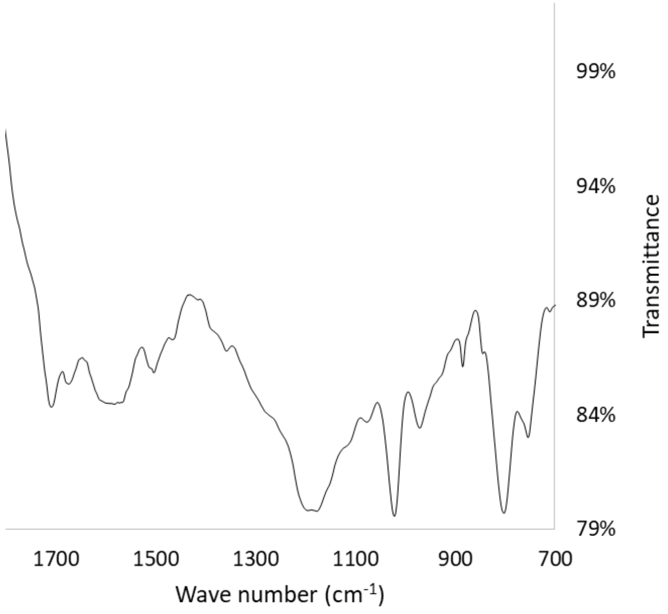

Supplementary Figure 16. IR spectrum of humins generated from D-glucose in the presence of 2-furfural

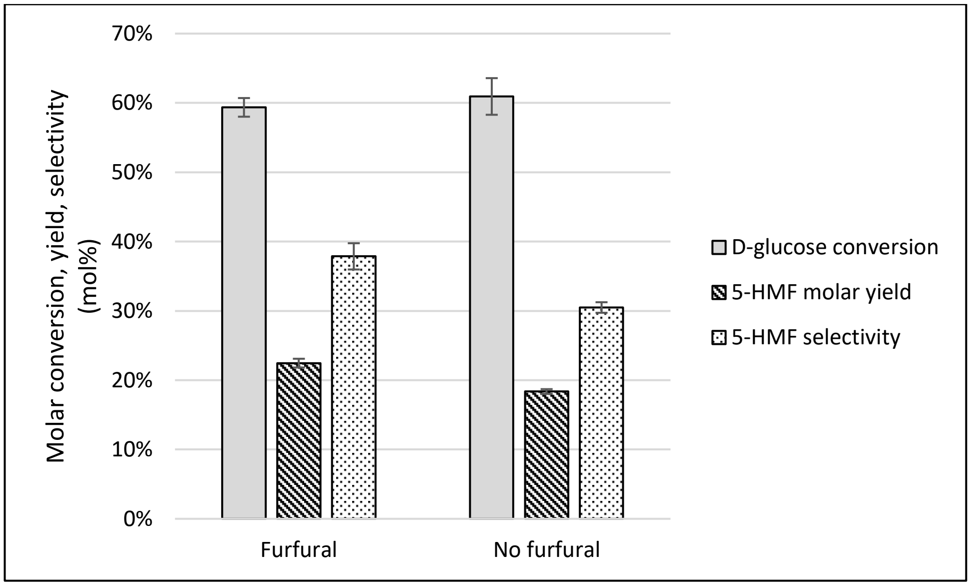

Supplementary Figure 17. Dehydration of D-glucose (0.56 mmol) to 5-HMF in the presence or absence of 2-furfural (2.6 mmol) in a mixture of choline chloride, boric acid and glycolic acid (4/1/1 mol) during 1 hour at 90°C (210 rpm).

2 Supplementary Tables

Supplementary Table 1. Absorbance (at 548 nm) of different mixtures of choline chloride (20 mmol), boric acid (5 mmol) and organic acid (5 mmol) containing 13 wt% water and 50 µL of thymol blue in ethanol (0.05 g/10 ml). To perform the tests with choline chloride/H_3BO_3 (2/1 mol) with or without hydrochloric acid, HCl (35.7%) was added to a 3 g mass of mixture to reach the desired concentration. Different amounts of water were added for each HCl concentration in order to keep the total water content at 13 wt%. 50 µL of thymol blue in ethanol (0.05 g/10 ml) were again added before homogenization and absorbance measurement.

Assay	Absorbance (548 nm)
ChCl/H3BO3 (2/1 mol) + HCl (0.4 mol/kg)	2.96
ChCl/H3BO3 (2/1 mol) + HCl (0.1 mol/kg)	2.94
ChCl/H3BO3 (2/1 mol) + HCl (0.01 mol/kg)	2.097
ChCl/H3BO3 (2/1 mol) + HCl (0.001 mol/kg)	1.564
ChCl/H3BO3 (2/1 mol) + HCl (0.0001 mol/kg)	1.508
ChCl/H3BO3 (2/1 mol)	1.47
ChCl/H3BO3/maleic acid (4/1/1)	2.059
ChCl/H3BO3/malic acid (4/1/1)	2.562
ChCl/H3BO3/glycolic acid (4/1/1)	2.422
ChCl/H3BO3/acetic acid (4/1/1)	0.337
ChCl/H3BO3/formic acid (4/1/1)	0.784
